# Impact of quitline services on tobacco cessation: an application of modern epidemiologic methods

**DOI:** 10.1093/aje/kwae292

**Published:** 2024-08-21

**Authors:** Ami E Sedani, Summer G Frank-Pearce, Sixia Chen, Jennifer D Peck, Janis E Campbell, Ann F Chou, Laura A Beebe

**Affiliations:** Department of Biostatistics and Epidemiology, Hudson College of Public Health, University of Oklahoma Health Sciences Center, Oklahoma City, OK 73104, United States; Division of Epidemiology and Social Sciences, Institute for Health and Equity, Medical College of Wisconsin, Milwaukee, WI 53226, United States; Department of Biostatistics and Epidemiology, Hudson College of Public Health, University of Oklahoma Health Sciences Center, Oklahoma City, OK 73104, United States; TSET Health Promotion Research Center, Stephenson Cancer Center, The University of Oklahoma Health Sciences Center, Oklahoma City, OK 73104, United States; Department of Biostatistics and Epidemiology, Hudson College of Public Health, University of Oklahoma Health Sciences Center, Oklahoma City, OK 73104, United States; Department of Biostatistics and Epidemiology, Hudson College of Public Health, University of Oklahoma Health Sciences Center, Oklahoma City, OK 73104, United States; Department of Biostatistics and Epidemiology, Hudson College of Public Health, University of Oklahoma Health Sciences Center, Oklahoma City, OK 73104, United States; Department of Family and Preventive Medicine, College of Medicine, University of Oklahoma Health Sciences Center, Oklahoma City, OK 73104, United States; Department of Biostatistics and Epidemiology, Hudson College of Public Health, University of Oklahoma Health Sciences Center, Oklahoma City, OK 73104, United States

**Keywords:** inverse probability weighting, quitlines, tobacco cessation, causal inference, selection bias

## Abstract

This study investigated the effectiveness of quitline service intensity (high vs low) on past 30-day tobacco abstinence at 7-months’ follow-up, using observational data from the Oklahoma Tobacco Helpline (OTH) between April 2020 and December 2021. To assess the impact of loss to follow-up and nonrandom treatment assignment, we fit the parameters of a marginal structural model to estimate inverse probability weights for censoring (IPCW), treatment (IPTW), and combined (IPCTW). The risk ratio (RR) was estimated using modified Poisson regression with robust variance estimator. Of the 4695 individuals included in the study, 64% received high-intensity cessation services, and 53% were lost to follow-up. Using the conventional complete case analysis (responders only), high-intensity cessation services were associated with abstinence (RR = 1.18; 95% CI, 1.04-1.34). The effect estimate was attenuated after accounting for censoring (RR = 1.14; 95% CI, 1.00-1.30). After adjusting for both baseline confounding and selection bias via IPTCW, high-intensity cessation services were associated with 1.23 times (95% CI, 1.08-1.41) the probability of abstinence compared to low-intensity services. Despite relatively high loss to follow-up, accounting for selection bias and confounding did not notably impact quit rates or the relationship between intensity of quitline services and tobacco cessation among OTH participants.

## Introduction

Over the past 50 years, the reduction in cigarette use has been considered one of the most significant public health successes and has helped shape epidemiologic concepts of causality.^1^^‑^^5^ However, tobacco use remains a leading cause of preventable disease and death.^6^^,^^7^ In 2020, approximately 1 in 5 US adults currently used tobacco, with higher rates persisting or even worsening among many historically marginalized and stigmatized populations.^8^^‑^^10^ Tobacco cessation quitlines (helplines) are a strategy which have proven effective in promoting cessation across diverse populations, providing accessible and evidence-based services without major barriers.^11^^‑^^13^ Therefore, they have the potential to reach socially, economically, and otherwise disadvantaged populations and are seen as an integral component of comprehensive tobacco control strategies.^14^^‑^^16^ Evaluating the effectiveness of state tobacco quitline interventions is essential for their continued success. The North American Quitline Consortium (NAQC) recommends conducting follow-up surveys with a random sample of participants 7 months after registration to measure tobacco abstinence or quit rates (ie, number of respondents reporting abstinence for 30 days or longer at the 7-month follow-up survey divided by the total number responding to the survey).^17^ However, loss to follow-up may introduce selection bias, especially as response rates on follow-up surveys have decreased over time (from 48% in 2010 to 37% in 2020), causing concerns regarding the validity of quitline outcomes.^18^ Selection bias in cohort studies may occur when retention is affected by both the treatment/intervention (ie, intensity of quitline services) and another factor (eg, socioeconomic status, age group) that is also associated with the outcome (ie, cessation). Valid estimates are vital to monitor progress and justify funding allocation for research and tobacco control programming.

Existing quitline evidence has been primarily derived from studies that are vulnerable to selection bias. This could explain the conflicting findings in studies regarding the effectiveness of higher-intensity tobacco cessation interventions.^15^^,^^19^^‑^^32^ Only a limited number of studies have specifically examined tobacco quitline services, which are not only evidence-based but also more accessible compared to clinic-based services that often require transportation and health insurance.^23^^‑^^25^^,^^28^ As a result, the majority of these studies may not be generalizable to populations that have the highest tobacco use rates and suffer the largest disparities (ie, type 2 selection bias).^33^ In contrast, data generated from quitline studies may generalize to the target population but be more susceptible to selection bias due to loss to follow-up, with a majority of the studies using either intent to treat (ITT) analyses or complete case (responder only) analyses.

Despite its importance, there is a paucity of available data assessing the impact of loss to follow-up, resulting from nonresponse to quitline follow-up surveys, on current conclusions.^34^^‑^^36^ Three studies attempted to address this by using additional efforts to track down participants who were initially lost to follow-up.^34^^‑^^36^ Findings were mixed, with one study finding nonresponders were more likely to still be smoking cigarettes,^34^ one reporting solely on differences in outcomes by the number of contact attempts,^35^ and another where nonresponders were not more likely to still be smoking cigarettes.^36^ These studies also varied in terms of follow-up times, outcome measurements, and target populations. Restricting the analysis to the uncensored data (only responders) in quitline follow-up studies could induce a noncausal association between exposure (in this case, intensity of quitline services [high vs low]) and outcome (tobacco abstinence).^37^^‑^^39^ This could pose a potential threat to internal validity due to collider-stratification bias (ie, collider bias),^33^^,^^40^ especially if retention is differential across social groups. Alternatively, loss to follow-up can be reflective of hard-to-reach populations (also referred to as hidden populations or overlooked and underserved communities).^40^

In this study, we aim to estimate the causal effect of high-intensity quitline services on 7-month tobacco abstinence (quit status) compared to low-intensity services, using observational data from the Oklahoma Tobacco Helpline (OTH). We use inverse probability weighting (IPW) to evaluate and address selection bias from censoring limitations in standard methods, as well as to control for baseline confounding. The application of modern epidemiologic methods like IPW remains limited in the applied epidemiologic and tobacco literature^41^^,^^42^ and nonexistent in quitline studies, specifically in the evaluation of the effectiveness. While current approaches have provided the tobacco quitline field important insights about quit rates and program outcomes, the extent to which selection bias has affected those conclusions remains largely unaddressed. This research is imperative as the field continues to refine the standards used to maintain and improve the effectiveness of quitline services. Therefore, findings will have implications for informing quitline service delivery and expansion.

## Methods

### Data source

At the time of this study, the OTH offered varying levels of evidence-based interventions (eg, behavioral coaching, nicotine replacement therapy [NRT], etc.) including single-call, multiple-call, individual services, and WebCoach programs. Eligibility for these programs was determined by an individual’s health insurance status (details are provided in Appendix S1). OTH data for this analysis were derived from 3 sources. First, the standardized intake survey administered at registration (baseline) via phone or online collected sociodemographic and tobacco use information. Although the registration intake survey was converted to allow online registration, not all questions were transferred. Nevertheless, since web registrations are increasing and accounted for 31% of the sample, these individuals were not excluded. However, this resulted in some variables having significant proportion missing, and thus not being included in this analysis (eg, educational attainment, chronic conditions, general health status, etc.). Second, the OTH services delivery database includes program enrollment and services received (number of calls completed and amount of NRT shipped) over time. Third, to assess effectiveness of services, a random sample of participants who completed at least 1 call or received at least 2 weeks of NRT, was selected each month for the standardized follow-up. Eligible individuals were sent a prenotification letter and contacted via email. A follow-up post card was sent to nonresponders. Approximately 12 attempts, within 2 weeks of the initial attempt, were made to reach the participant by phone.

### Study design and study population

**Figure 1:**
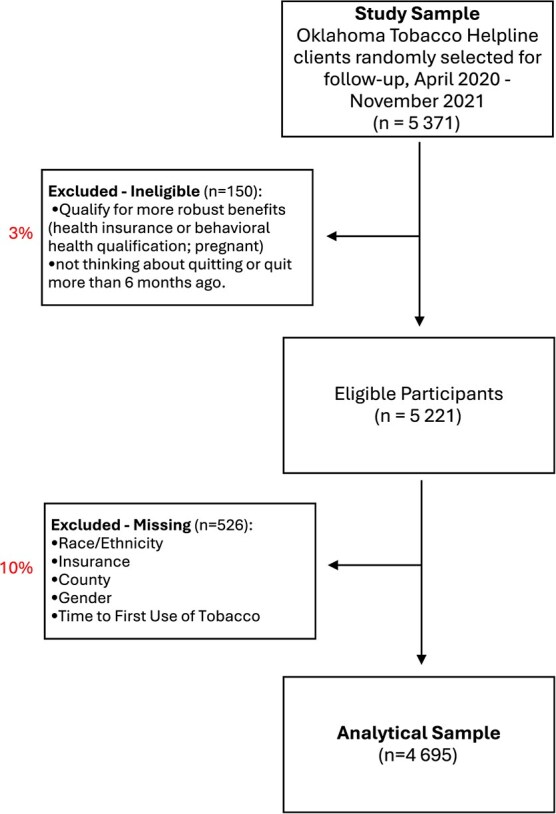
Flowchart of sample selection from the Oklahoma tobacco helpline (OTH).

We designed this cohort study using secondary observational data from OTH.^43^ The overall OTH evaluation and secondary analyses of OTH data were approved by the University of Oklahoma Health Sciences Center IRB (#2616). This study included only individuals who registered and were selected for follow-up between April 1, 2020, and November 30, 2021 (*n* = 5371). Additional study population details are provided in Appendix S1. Total sample size for analysis was 4695 (Figure 1).

### Measures

#### Intervention

While OTH clients registering for quitline services are assigned a program, the intensity of quitline services received varies depending on engagement (ie, interaction with the quitline). The intensity of intervention (quitline cessation services) was structured according to number of completed calls and number of weeks of NRT supplied by OTH (Table S1). All levels of intensity of services could also include web and/or text and/or email support. The typical duration of quitline intervention is up to 6 weeks. Each individual was “assigned” to an intervention arm based on their utilization of services. To aid in interpretability and analyses, quitline service intensity levels were collapsed into binary categories (high vs low), where low is defined as at least 1 completed call and no NRT, or no completed call and 2 weeks of NRT supplied, and high is defined as one of a number of different combinations of greater NRT and more completed calls.^44^^,^^45^

#### Outcome

The primary outcome was quit status, defined as self-reported past 30-day point-prevalence abstinence (PPA) from tobacco products measured at 7 months postregistration. Quit status was derived from a “no” response by participants to the question, “Have you smoked any cigarettes or used tobacco, even a puff or a pinch, in the last 30 days (does not include use of tobacco in American Indian ceremonies).” This is in alignment with the NAQC’s benchmark used to assess quality of quitlines.^17^ Alternative products (eg, electronic cigarettes [with or without nicotine] and other vaping devices) are not included in this definition.^46^

#### Covariates

The variables included in our study are commonly found in quitline data sets, and their selection is based on thorough research findings.^47^ The following baseline demographic and tobacco use covariates were used for our analyses: age (in years), gender, self-designated race/ethnicity, income, health insurance status, geographic residence, tobacco products used, time after waking to first tobacco use, and registration mode.

### Analysis

Descriptive statistics were compiled to examine baseline characteristics of the study sample. Roughly 7% (*n* = 363) of individuals were missing income data. Therefore, multiple imputation using logistic regression method was utilized for missing values on the income variable, under the missing at random (MAR) assumption.^48^^,^^49^ Simulated complete data sets were generated via regression imputation, and we employed the analytical procedure in SAS to combine the results using 25 imputations. Variables included in the imputation procedure for income included primary outcome, age, gender, race/ethnicity, geographical residence, health insurance status, tobacco products used, and time after waking to first tobacco use.^50^ To address small cell sizes, categories were collapsed for self-designated race/ethnicity, and health insurance plan. Frequencies, percentages, and corresponding 95% confidence intervals (CIs) were calculated and reported. We fit the parameters of a marginal structural model in a 2-step process: (1) estimating inverse probability weights for censoring (ie, attrition) and treatment (ie, intervention), and then (2) employing the unstabilized weights in a marginal structural model to estimate the parameters of interest.

#### Estimating censoring weights (IPCW)

We used inverse probability of censoring weighting (IPCW) to address potential selection bias induced by missing values in the outcome variable. Reasons for censoring were explored to determine if we needed to develop different analytical models to distinguish between different types of selective attrition in analysis.^42^ Censoring applied to refusals, those who were deceased, unreachable (eg, disconnected/non-working number), or “other.” The majority of participants was unreachable (*n* = 1775; 71.78%), and mortality accounted for less than 0.57% (*n* = 14) of the attrition. Therefore, we did not consider different types of attrition in the analysis. Individuals were categorized as responders if their outcome variable was obtained and nonresponders if they were lost to follow-up. Weights for IPCW is estimated as 1/[Pr(C = 0 | A, Z)], where *Z* is a set of baseline covariates, *A* is the binary intervention, and *C* = 0 for uncensored (responders). Subject matter knowledge was used to identify variables for the IPCW model that we thought were likely to influence censoring and tobacco abstinence.^51^^‑^^55^ Our assumptions about the causal structure of our data are visualized in a directed acyclic graph (DAG) in Figure S1. Intervention group, age, race/ethnicity, health insurance plan, and geographical residence were selected to estimate ICPW using a logistic regression model**.**

#### Estimating treatment weights (IPTW)

To estimate the causal treatment effects and balance confounders, a similar process was used to estimate the inverse probability of treatment weighting (IPTW), a type of propensity score analysis. Weights are based on results from a treatment selection model. Therefore, the dependent variable was treatment (high- vs low-intensity services). Weights for IPTW is estimated as Pr[A = 1 |Z], where *Z* is a set of baseline covariates and *A* is the binary intervention group. Subject matter knowledge was used to identify a minimally sufficient set of variables likely associated with the intervention and tobacco abstinence.^51^^‑^^55^ Age, health insurance plan, and income were used to estimate IPTW using a logistic regression model.

#### IPW marginal structural models

The final weight used in the causal model was the inverse probability of treatment and censoring weight (IPTCW), which is equal to the product of weights for confounding adjustment (ie, IPTW) and selection bias adjustment (ie, IPCW).^55^^,^^56^ Our assumptions about the causal structure of our data are visualized in a DAG in Figure S2. Next, the distribution of the weights was summarized, and covariate balance was visually inspected. We then fit marginal structural models to estimate the effects of quitline service intensity on tobacco cessation using IPTCW as an analytic weight. The risk ratio (RR) was estimated using modified Poisson regression with a robust variance estimator using the SAS procedure PROC GENMOD.^57^^‑^^59^ We also calculated the quit rates using complete case data (unadjusted, responders only) and using IPCW, IPTW, and IPTCW weights. All analyses were performed in SAS version 9.4 (SAS Institute, Inc., Cary, NC, USA), and a *P* value of <.05 was considered statistically significant.

#### Sensitivity analyses

To check the robustness of our estimates, first, 3 IPW methods were explored: stabilizing, weight truncation (trimming), and a survey calibration technique. The RR estimates from the models adjusted with unstabilized and stabilized weights provided identical point estimates; therefore, unstabilized results were reported (Table S2). Model misspecification was also explored by including all measured baseline covariates except for registration mode and tobacco products. The RR estimates provided identical point estimates up until the thousandths and ten-thousandths decimal places (Table S3).

## Results

### Participant characteristics

Of the 4695 individuals included in the study, 63.69% received high-intensity cessation services. At baseline, mean age of participants was 48.29 (± 14.95; range: 18-94), and more than half of the individuals reported their gender as female (56.04%). The majority of participants self-designated their race/ethnicity as White (70.44%), followed by American Indian or Alaskan Native (9.50%), and Black or African American (8.20%). Approximately 30.84% of individuals had an income of less than $10 000, and 32.33% were uninsured. More than half of the sample (62.26%) lived in a metropolitan county. Consistent with typical state quitline populations,^60^ cigarettes were the most commonly used tobacco product (78.06%), and 10.99% of participants reported using 2 or more tobacco products. Differences in the distributions of these variables between participants in each intervention group are presented in Table 1. Individuals in the low-intensity intervention group had a lower mean age, had higher annual income, and were more likely to have private health insurance and to have registered online.

**Table 1:** Comparison of baseline descriptive characteristics by cessation service-intensity prior to weighting, Oklahoma, April 2020-December 2021 (*n* = 4695).^a^

	**Total**	**Low intensity**	**High intensity**	***P***
	4695	1704 (36.31)	2991 (63.69)	
Mean age ± SD	48.29 ± 14.95	45.47 ± 14.70	49.89 ± 14.85	<.0001^*^
Age category				<.0001^*^
18-24	232 (4.94)	114 (6.69)	118 (3.95)	
25-34	805 (17.15)	357 (20.95)	448 (14.98)	
35-44	954 (20.32)	382 (22.42)	572 (19.12)	
45-54	916 (19.51)	338 (19.84)	578 (19.32)	
55-64	1071 (22.81)	327 (19.19)	744 (24.87)	
65+	717 (15.27)	186 (10.92)	531 (17.75)	
Gender				0.1806
Female	2631 (56.04)	933 (54.75)	1698 (56.77)	
Male	2064 (43.96)	771 (45.25)	1293 (43.23)	
Self-designated race/ethnicity				0.0887
NH White	3307 (70.44)	1220 (71.60)	2087 (69.78)	
NH Black or African American	385 (8.20)	118 (6.92)	267 (8.93)	
NH AI/AN	446 (9.50)	172 (10.09)	274 (9.16)	
NH other	354 (7.54)	35 (2.05)	65 (2.17)	
Hispanic	203 (4.32)	63 (3.70)	140 (4.68)	
Annual income				<.0001*
Less than $10 000	1462 (31.14)	494 (28.99)	968 (32.36)	
$10 000-14 999	770 (16.4)	254 (14.91)	516 (17.25)	
$15 000-19 999	531 (11.31)	161 (9.45)	370 (12.37)	
$20 000-24 999	448 (9.54)	168 (9.86)	280 (9.36)	
$25 000-34 999	519 (11.05)	213 (12.50)	306 (10.23)	
$35 000-49 999	472 (10.05)	184 (10.80)	288 (9.63)	
$50 000 or more	493 (10.50)	230 (13.50)	263 (8.79)	
Health insurance plan				<.0001*
Private	1008 (21.47)	443 (26.00)	565 (18.89)	
Medicaid	923 (19.66)	300 (17.61)	623 (20.83)	
Medicare	1019 (21.70)	268 (15.73)	751 (25.11)	
Other public	227 (4.83)	90 (5.28)	137 (4.58)	
Uninsured	1518 (32.33)	603 (35.39)	915 (30.59)	
Geographic residence				0.5006
Rural	755 (16.08)	278 (16.31)	477 (15.95)	
Micropolitan	1017 (21.66)	383 (22.48)	634 (21.20)	
Metropolitan	2923 (62.26)	1043 (61.21)	1880 (62.86)	
Time to first tobacco use				0.2236
Within 5 minutes	2199 (46.84)	776 (45.54)	1423 (47.58)	
6-30 minutes	1663 (35.42)	611 (35.86)	1052 (35.17)	
31-60 minutes	494 (10.52)	198 (11.62)	296 (9.90)	
More than 60 minutes	339 (7.22)	119 (6.98)	220 (7.36)	
Tobacco products				0.0568
Multiple tobacco products	516 (10.99)	197 (11.56)	319 (10.67)	
Cigarette	3665 (78.06)	1296 (76.06)	2369 (79.20)	
Smokeless	353 (7.52)	142 (8.33)	211 (7.05)	
Other	161 (3.43)	69 (4.05)	92 (3.08)	
Registration mode				<.0001^*^
Phone	3017 (64.26)	832 (48.83)	2185 (73.05)	
Online	1269 (27.03)	730 (42.84)	539 (18.02)	
Referral/other	409 (8.71)	142 (8.33)	267 (8.93)	

Abbreviations: SD, standard deviation; NH, non-Hispanic.

^a^Data are presented as no. (%) unless otherwise indicated. χ^2^ test and *t* test were used to calculate the *P* values. Asterisks (*) indicates statistically significant at ≤.05

### Characteristics by censoring

Approximately 47.33% (*n* = 2222) of the analytical sample remained at follow-up (responders), constituting the analytic sample used in the complete case analysis. Table 2 presents differences in distributions of covariates between responders and nonresponders. Accordingly, nonresponders had a lower mean age, and were more likely to be non-Hispanic (NH) White, uninsured, from a rural area, first use tobacco within 5 minutes after waking, and receive low-intensity cessation services. Hence, if using conventional complete-case analysis, the study sample would overrepresent those who were older and in the high-intensity group and would underrepresent individuals who are NH White, uninsured, live in rural county, and have higher tobacco dependence (reported first using tobacco within 5 minutes of waking).

**Table 2:** Comparison of descriptive characteristics of OTH responders and nonresponders (*n* = 4695), Oklahoma, 2020-2021.^a^

**Baseline covariates**	**Responders (*n* = 2222; [47.33%])**	**Non-responders (*n* = 2473; [52.67%])**	***P***
Mean age ± SD	50.13 (±14.74)	46.63 (±14.94)	<.0001^*^
Age category			<.0001^*^
18-24	82 (3.69)	150 (6.07)	
25-34	319 (14.36)	486 (19.65)	
35-44	430 (19.35)	524 (21.19)	
45-54	432 (19.44)	484 (19.57)	
55-64	568 (25.56)	503 (20.34)	
65+	391 (17.60)	326 (13.18)	
Gender			0.1012
Female	1273 (57.29)	1358 (54.91)	
Male	949 (42.71)	1115 (45.09)	
Self-designated race/ethnicity			0.0002^*^
NH White	1512 (68.05)	1795 (72.58)	
NH Black	222 (9.99)	163 (6.59)	
NH AI/AN	216 (9.72)	230 (9.30)	
NH multiple races or other	178 (8.01)	176 (7.12)	
Hispanic	94 (4.23)	109 (4.41)	
Annual income			0.6554
Less than $10 000	673 (30.29)	789 (31.90)	
$10 000-14 999	354 (15.93)	416 (16.82)	
$15 000-19 999	267 (12.02)	264 (10.68)	
$20 000-24 999	212 (9.54)	236 (9.54)	
$25 000-34 999	246 (11.07)	273 (11.04)	
$35 000-49 999	229 (10.31)	243 (9.83)	
$50 000 or More	241 (10.85)	252 (10.19)	
Health insurance plan			<.0001^*^
Private	496 (22.32)	512 (20.70)	
Medicaid	437 (19.67)	486 (19.65)	
Medicare	533 (23.99)	486 (19.65)	
Other public	123 (5.54)	104 (4.21)	
Uninsured	633 (28.49)	885 (35.79)	
Geographic residence			0.0238^*^
Rural	323 (14.54)	432 (17.47)	
Micropolitan	492 (22.14)	525 (21.23)	
Metropolitan	1407 (63.32)	1516 (61.30)	
Time to first tobacco use			0.0063^*^
Within 5 minutes	981 (44.15)	1218 (49.25)	
6-30 minutes	828 (37.26)	835 (33.76)	
31-60 minutes	247 (11.12)	247 (9.99)	
More than 60 minutes	166 (7.47)	173 (7.00)	
Tobacco product			0.0904
Multiple tobacco products	221 (9.95)	295 (11.93)	
Cigarettes	1742 (78.4)	1923 (77.76)	
Smokeless	175 (7.88)	178 (7.20)	
Other	84 (3.78)	77 (3.11)	
Intervention intensity			<.0001^*^
Low	683 (30.74)	1021 (41.29)	
High	1539 (69.26)	1452 (58.71)	
Registration mode			0.1159
Phone	1427 (64.22)	1590 (64.29)	
Online	583 (26.24)	686 (27.74)	
Referral/other	212 (9.54)	197 (7.97)	

Abbreviations: SD, standard deviation; NH, non-Hispanic.

^a^Data are presented as no. (%) unless otherwise indicated. χ^2^ test and t-test were used to calculate the *P* values

^*^Statistically significant at ≤0.05

^“^Other public” health insurance category includes Indian Health Services (IHS) and Veteran.

### Assessing covariate balance and weight distribution

Table S4 presents distributions of weights as well as effect size estimate. When evaluating covariate balance before and after the application of the censoring weights, weighted covariate distributions were comparable to the distributions observed in the total unweighted sample (Table S5). Similarly, after IPTW weighting, covariate distributions became similar between the 2 intervention groups (ie, CI overlap), thus indicating improvement in covariate balance (Table S6).^61^ For variables with small categories, slight differences did persist. For example, differences in age category can be observed by intervention group for categories with the youngest and oldest participants; however, this is a smaller difference than that observed among the groups before weighting.

### Quitline service intensity and tobacco abstinence

The overall quit rate varied slightly by method from 33.62% when weighted for treatment assignment to 34.92% when weighted for selection bias (Figure S3). The conventional complete case analysis (unadjusted, responders only) resulted in a quit rate of 34.88%. Compared to low-intensity cessation services intervention group, high-intensity cessation services were associated with 1.18 times (95% CI, 1.04-1.34) the probability of 30-day point-prevalence abstinence at 7 months (*P = .*0130; Table 3). After weighting using IPCW, the RR estimate was attenuated but still showed an association. Thus, after accounting for censoring compared to low-intensity cessation services, high-intensity cessation services were associated with 1.14 times (95% CI, 1.00, 1.30) the probability of abstinence (*P = .*0432). After adjusting for baseline confounding using IPTW, high-intensity cessation services were associated with 1.27 times (95% CI, 1.11-1.45) the probability of abstinence compared to low-intensity cessation services (*P = .*0006). Accounting for baseline confounding and selection bias via IPTCW did not substantially change the effect estimates; therefore, high-intensity cessation services were associated with 1.23 times (95% CI, 1.08-1.41) the probability of abstinence (*P = .*0025) compared to low-intensity services.

**Table 3:** Results of analysis methods estimating risk ratio of tobacco abstinence for high intensity quitline services, Oklahoma, 2020-2021.^a^

	**Risk ratio**	**95% CI**	***P***
**Unweighted estimates**			
Complete case (Unadjusted)	1.18	1.04-1.34	0.0130
**Weighted estimates**			
Adjusting for selection bias using IPCW^a^	1.14	1.00-1.30	0.0432
Adjusting for confounding using IPTW^b^	1.27	1.11-1.45	0.0006
Adjusting for both confounding and selection bias using IPTCW^c^	1.23	1.08-1.41	0.0025

^a^Inverse Probability Censoring Weight (IPCW) created with adjustment for intervention, age, race/ethnicity, health insurance plan, and geographic residence.

^b^Inverse Probability of Treatment Weight (IPTW) created with adjustment for age, health insurance plan, and income.

^c^Inverse Probability of Treatment and Censoring Weight (IPTCW) is IPCW^*^IPTW.

To facilitate comparison to conventional analyses, quit rates by participant characteristics were examined before and after weighting (Table S7). Although the weighted quit rates among some covariate categories were larger than unweighted quit rates, those CIs overlapped substantially. Specifically, the weighted quit rates did not appear to differ meaningfully by participant characteristics compared to those from complete case analyses.

## Discussion

Addressing selection bias is of increased importance as quitline follow-up response rates have been gradually decreasing, and achieving target response rates requires resources that are not always available within quitline budgets. Many quitline studies acknowledge a low response rate for follow-up surveys as a significant limitation. It is important to understand the implications of low response rates for quitline evaluation, as they may compromise results and subsequent conclusions. Quitline studies are advantageous for disparities research. Our results bridge a gap in the literature regarding the extent to which selection bias affects the evaluation of a state tobacco quitline and revisits the causal association between quitline service intensity and tobacco cessation. Our study demonstrated that significant loss to follow-up (>50%) had minimal impact on quit rates and the effect estimates comparing high-intensity quitline cessation service to low-intensity. Thus, applying modern causal methods indicates that results based on conventional complete-case analyses were only modestly attenuated by selection bias and baseline confounding.

Nonresponse resulting from loss to follow-up can be troublesome due to its potential to introduce selection bias. The extent to which the overall follow-up rates in a study are associated with selection bias has been a subject of controversy.^62^ From April 2020 through November 2021, the OTH response rate was 47%, with a lower rate among the group of individuals receiving low intensity services compared to the high intensity group (40% vs 52%, respectively). Our analyses reveal some systematic differences between those who were lost to follow-up and participants that were retained. We found that nonresponders were more likely to be younger, self-designated NH White, uninsured, living in a rural residence, to have higher tobacco dependence (based on time to first tobacco use), and to be in the low-intensity intervention group. Many of these differences were also strongly associated with the intervention group (high- vs low-intensity), where low-intensity group members were more likely to be younger, uninsured, and to have a higher income compared to the high intensity group. However, our findings provide evidence to suggest that despite differential loss to follow-up, conditioning on a suspected collider did not markedly attenuate the relationship between exposure/intervention (quitline services) and outcome (tobacco abstinence) in our analytical sample.^63^

Several other studies that have looked at the effect of nonparticipation or attrition on estimates using IPW methods have also found minimal impact on estimates.^64^^‑^^70^ Specifically in literature regarding nonresponse to quitline follow-up surveys, Tomson et al expended additional efforts to reach nonresponders and found that they were not more likely to still be using tobacco.^36^ Fu et al also did not find a difference in outcomes by intervention groups when using a model-based likelihood method for missing data (expectation–maximization algorithm).^71^ Our study findings, along with those of the referenced studies, add to the evidence base suggesting that this type of bias may often be slight and offset by adjustment for confounders that are selection factors.^72^^‑^^75^ Other possible explanations for the absence of evidence for selection bias in the presence of disproportionate response rates by quitline participant characteristics may be (1) that quit rates did not differ in these under- and overrepresented groups as anticipated, or (2) that bias of similar magnitude was introduced in opposing directions which counteracted the overall impact on the corrected point estimate. Although we found negligible selection bias for the association between quitline service intensity and cessation, we cannot be sure that these results can be transported to other populations, or that it is not the result of unmeasured confounding or residual confounding by indication. It is important to assess the probability and magnitude of selection bias on a case-by-case basis to determine the degree to which loss to follow-up impacts the internal validity of the relationship of interest, as opposed to influencing the external validity of results.^76^^‑^^78^ Continued collection and monitoring of predictors of loss to follow-up is important to determine differences by availability of outcome data to further understand the factors associated with the potential bias, and to assess the quality of reported results.

### Strengths and limitations

There are several key limitations related to the data source and causal framework that warrant consideration when interpreting results of this study. Findings should only be interpreted causally under the causal inference assumptions of exchangeability, consistency, positivity, and correct model specification. Unfortunately, conditional exchangeability over censoring may not be fully attainable with IPCW due to the absence of key time-varying prognostic factors in our data set. Although robust, our approach still relies on the same assumptions as standard methods (model misspecification, unmeasured confounding, and information bias). First, we cannot rule out model misspecification, despite sensitivity analyses resulting in similar effect sizes. Model specification is also dependent on (accurate) variables in the database. Second, self-reported variables may be more susceptible to misclassification (information bias). The outcome of interest was based on self-report and was not biochemically verified and consequently may be an overestimation of abstinence.^79^ The validity of measurements can be related to interaction with an interviewer (eg, social desirability bias) even under optimal reporting and interview conditions, with some studies detecting variability between interviewers. However, self-reported tobacco outcomes are considered accurate in most cessation studies; biochemical verification is not considered necessary when data collection is done by mail, telephone, or internet.^80^^‑^^83^ Although the intervention was not self-reported, it is important to note that it may still be susceptible to misclassification. For example, the amount of NRT in our operationalization of intensity is based on the amount of NRT shipped to participants and may not accurately reflect the amount of NRT actually used by participants. We do not suspect significant misclassification among most covariates since they are demographic variables; however, income and time to first use of tobacco after waking may be impacted. Individuals may underestimate time to first use of tobacco after waking, since this may be habitual, repetitious, and almost unconscious; however, by giving participants categorical response options, only those consuming their tobacco close to category borders will need to contemplate which answer best fits them. Third, as in any observational study, unmeasured confounding cannot be completely ruled out. There are a few confounders that we hypothesize are important to the causal relationship but were not measured in the current study, including genetic predispositions, behavioral factors (eg, externalizing symptoms), and environmental factors. Lastly, given that the study was conducted during the COVID-19 pandemic, the findings may be influenced by the unique circumstances of the pandemic (eg, increased stress, differing access to tobacco products, and heightened fear regarding tobacco use and infection).

In light of these limitations, this study contributes to current literature in a number of ways. Randomized trials are often preferred; however, they are not feasible for all causal questions and are often costly. The successful use of appropriate methodology allows estimation of the causal effect of interventions from observational data, similarly to results of a randomized trial. Thus, this provides the ability to generate evidence to inform public health initiatives. Second, clinical trials have strong internal validity but often lack external validity (generalizability) and thus suffer from an efficacy-effectiveness gap. This study used data from a community-based quitline, and in most cases, quitlines are considered effectiveness trials because they are conducted within the context of operational quitlines, under real-world conditions. Real-world samples have been suspected to result in greater public health impact.^84^

## Conclusion

This study used modern epidemiologic methods to estimate a causal effect from observational data. Although others have shown structurally and empirically that differential participation alone does not result in substantial selection bias, many studies continue to make unsubstantiated claims about potential selection bias based solely on differences in participation rates among the exposure and outcome groups. Furthermore, we show that despite differences in the distribution of characteristics between responders and nonresponders (lost to follow-up), quit rates measured at the 7-month follow-up were not impacted. The impact of selection bias on an association depends on the strength of relationship of censoring with the study exposure and outcome; therefore, results should be replicated in relation to other state quitlines, and quitlines should continue to monitor the potential for selection bias.

## Supplementary Material

Web_Material_kwae292

## Data Availability

Data will be made available from the study Principal Investigator (Dr. Laura Beebe) upon reasonable request.
