# Correction to: Impact of quitline services on tobacco cessation: an application of modern epidemiologic methods

**DOI:** 10.1093/aje/kwag207

**Published:** 2026-09-28

This is a correction to: Ami E Sedani, Summer G Frank-Pearce, Sixia Chen, Jennifer D Peck, Janis E Campbell, Ann F Chou, Laura A Beebe, Impact of quitline services on tobacco cessation: an application of modern epidemiologic methods, *American Journal of Epidemiology*, Volume 194, Issue 5, May 2025, Pages 1322–1331, https://doi.org/10.1093/aje/kwae292

In the originally published version of this manuscript, the supplementary data file was erroneous and did not pertain to this article.

The correct supplementary data file is included here.

This error has been corrected only in this correction notice to preserve the version of record.

## Supplementary Material

Web_Material_kwag207

