# Supplementary material for: Impact of quitline services on tobacco cessation: an application of modern epidemiologic methods
Source: Am J Epidemiol. 2024 Aug 21;194(5):1322–31. doi: 10.1093/aje/kwae292 (PMC12055460; doi:10.1093/aje/kwae292)
Supplement: Web_Material_kwae292 [file web_material_kwae292.docx]

**Supplementary Material**

Potential Biases in Test-Negative Design Studies of COVID-19 Vaccine Effectiveness Arising from the Inclusion of Asymptomatic Individuals

Edgar Ortiz-Brizuela, Mabel Carabali, Cong Jiang, Joanna Merckx, Denis Talbot, Mireille E. Schnitzer.

Table of Contents

[Supplementary Text 2](#_Toc166931395)

[Appendix S1: A brief overview of causal directed acyclic graph theory and terminology. 2](#_Toc166931396)

[Appendix S2: Identifiability of causal effects under outcome-dependent sample selection. 4](#_Toc166931397)

[Supplementary Tables 7](#_Toc166931398)

[Table S1: Data generation process for the DAG-guided simulation study. 7](#_Toc166931399)

[Table S2: Bias in odds ratio (OR) estimates for symptomatic COVID-19 ($\boldsymbol{ORCOVID}$) and SARS-CoV-2 infection ($\boldsymbol{ORinfect}$) relative to their target parameters ($\boldsymbol{RRCOVID}$ and $\boldsymbol{RRinfect}$, respectively) after individually varying the strength of one selected parameters (assuming perfect SARS-CoV-2 diagnostic tests). 10](#_Toc166931400)

[Table S3: Bias in odds ratio (OR) estimates for symptomatic COVID-19 ($\boldsymbol{ORCOVID}$) and SARS-CoV-2 infection ($\boldsymbol{ORinfect}$) relative to their target parameters ($\boldsymbol{RRCOVID}$ and $\boldsymbol{RRinfect}$, respectively) after simultaneously varying the strength of two or three selected parameters (assuming perfect SARS-CoV-2 diagnostic tests). 13](#_Toc166931401)

[Table S4: Bias in odds ratio (OR) estimates for measured symptomatic COVID-19 ($\boldsymbol{ORCOVID}$) and measured SARS-CoV-2 infection ($\boldsymbol{ORinfect}$) relative to their target parameters ($\boldsymbol{RRCOVID}$ and $\boldsymbol{RRinfect}$, respectively) after individually varying the strength of selected parameters (assuming symptomatic individuals are tested exclusively with NAATs and asymptomatic individuals are tested exclusively with antigen tests). 15](#_Toc166931402)

[Table S5: Bias in odds ratio (OR) estimates for measured symptomatic COVID-19 ($\boldsymbol{ORCOVID}$) and measured SARS-CoV-2 infection ($\boldsymbol{ORinfect}$) relative to their target parameters ($\boldsymbol{RRCOVID}$ and $\boldsymbol{RRinfect}$, respectively) after individually varying the strength of two or three selected parameters (assuming symptomatic individuals are tested exclusively with NAATs and asymptomatic individuals are tested exclusively with antigen tests). 18](#_Toc166931403)

[Supplementary Figures 20](#_Toc166931404)

[Figure S1: Causal directed acyclic graphs for "alternative" test-negative design studies of COVID-19 vaccine effectiveness - highlighting the remaining open paths between vaccination status $\mathbf{(}\boldsymbol{V}\mathbf{)}$ and the outcome infection ($\boldsymbol{I}$) after restricting the study sample to tested individuals ($\boldsymbol{T}\mathbf{=1}$). 20](#_Toc166931405)

[Appendix References 22](#_Toc166931406)

# Supplementary Text

## **Appendix S1**: A brief overview of causal directed acyclic graph theory and terminology.

A causal Directed Acyclic Graph (DAG) is a collection of "nodes" representing random variables, with their causal (temporal) relationships illustrated by unidirectional (directed) arrows, also known as "arcs."[^1-3^](#_ENREF_1) In a DAG, no variable can be caused by itself, either directly or indirectly through other variables, making it "acyclic." When an arc connects two nodes, the variable from which it originates is called the "parent," and the variable to which it points is referred to as its "child." Moreover, a "path" is any sequence of arcs connecting two nodes, regardless of their direction. A "directed path" is a path where all arcs point in the same direction, representing a causal path. In contrast, non-directed paths between two variables that lead to their association (as explained below) are called "biasing paths". For example, the path $\boldsymbol{A\leftarrow B\to C}$ is a non-directed (and biasing) path between $\boldsymbol{A}$ and $\boldsymbol{C}$, also known as a "backdoor path" because it starts with an arc pointing to $\boldsymbol{A}$ and ends with an arrow pointing to $\boldsymbol{C}$.

In a directed path, variables in proximal positions are called "ancestors" of those in distal positions, and those in distal positions are "descendants" of those in proximal positions. Furthermore, nodes in any path can be classified as "colliders" or "non-colliders." A "collider" in a path is a node with its preceding and subsequent arcs pointing at it (e.g., $\boldsymbol{B}$ is a collider in the path $\boldsymbol{A\to B\leftarrow C}$). In contrast, a "non-collider" can be classified as a "mediator" if the variable occupies an intermediate position in the directed path, or as a "fork" if the preceding and subsequent arcs emerge from it. For example, $\boldsymbol{B}$ is a mediator in the path $\boldsymbol{A\to B\to C}$, while $\boldsymbol{B}$ is a fork in the path $\boldsymbol{A\leftarrow B\to C}$.

One of the main benefits of DAGs is that they allow users to evaluate potential sources of association between variables and, consequently, to identify possible sources of bias. In general, according to DAG theory, two nodes are expected to be statistically associated if any path between them is "open" (in this case, variables are categorized as "d-connected," otherwise they are considered "d-separated").[^1^](#_ENREF_1)^,^[^3^](#_ENREF_3) A path between two nodes is "closed" if 1) it contains a collider (for which the analysis has not been conditioned on, nor on its descendants), or 2) if we condition our analysis on any non-collider within the path.[^1^](#_ENREF_1) For example, "confounding" (i.e., the presence of open backdoor paths) or "collider bias" (i.e., opening a non-causal path by conditioning on a collider or its descendants) are examples of sources of non-causal (biased) associations between two variables that can be detected using DAGs.

Importantly, in order to attribute any found association between two variables in the DAG solely to open paths, we must assume that the DAG is missing no variable that affects two or more variables in it, that no variable that affects selection or is used for stratification that may be a collider is missing, and that there is no measurement or random error that could explain the association.[^3^](#_ENREF_3) We refer readers to other sources for a more comprehensive yet gentle introduction to DAG theory.[^1-3^](#_ENREF_1)

## **Appendix S2**: Identifiability of causal effects under outcome-dependent sample selection.

In studies where sampling depends on outcome status, such as traditional case-control studies or the test-negative design (TND), the risk of the outcome, the risk ratio, and the risk difference are subject to bias.[^4^](#_ENREF_4)^,^[^5^](#_ENREF_5) Didelez et al.[^6^](#_ENREF_6) outlined two conditions that can be evaluated graphically using directed acyclic graphs (DAGs) necessary for both identifying and generalizing the conditional causal odds ratio (OR) in such circumstances:

1. The first condition — needed to identify the conditional causal OR within the study sample — requires that no backdoor path remains open between exposure and outcome (see **Appendix S1** for further details on DAG theory). This means that in the classical TND, after conditioning on health and healthcare-seeking behaviors (HSBs ($H$)) and the set of measured confounders ($C$), no backdoor path must remain open between the node vaccination status ($V$) and all components of the outcome medically attended and laboratory-confirmed symptomatic COVID-19 ($I\cdot S\cdot T$). This condition can be achieved within the classical TND by restricting the study sample to healthcare seekers ($H=1$) and conditioning on the set of measured confounders ($C$) during the analysis.

On the other hand, for this condition to be met in the alternative TND, no backdoor path must remain open between the vaccination status ($V$) and infection ($I$) after conditioning on HSBs ($H$) and the set of measured confounders ($C$). However, because the study sample is not limited to individuals with HSBs, it may not be possible to achieve this condition in the alternative TND. Therefore, while the conditional causal OR for medically attended and laboratory-confirmed symptomatic infection can be identified in the classical TND, the conditional causal OR for infection may remain unidentified in the alternative TND (our manuscript discusses numerous instances where conditional exchangeability on exposure is violated in the alternative TND).

1. The second condition — needed to generalize the conditional causal OR from the study sample to the entire population — requires the node for exposure status to be independent of the node selection, after conditioning on the outcome and a set of measured variables. In the classical TND, this means that the node representing an individual's vaccination status ($V$) must be independent of the node representing selection into the study ($Sel$, see DAG below), after conditioning on the outcome medically attended and laboratory-confirmed symptomatic COVID-19 ($I\cdot S\cdot T$), HSBs ($H$), and the set of measured confounders ($C$). Using notation: $V⫫Sel | (I\cdot S\cdot T, H, C)$. In the alternative TND, this condition translates into the node representing an individual's vaccination status ($V$) being independent of the node selection into the study ($Sel$), after conditioning on infection ($I$), HSBs ($H$), and the set of measured confounders ($C$). Using notation: $V ⫫ Sel | (I,H,C)$. In other words, based on our assumed DAG, only the conditional causal OR for medically attended and laboratory-confirmed symptomatic infection can be identified and generalized in the classical TND. Conversely, in the alternative TND, the conditional causal OR for infection may not be identifiable or generalizable to the broader population.


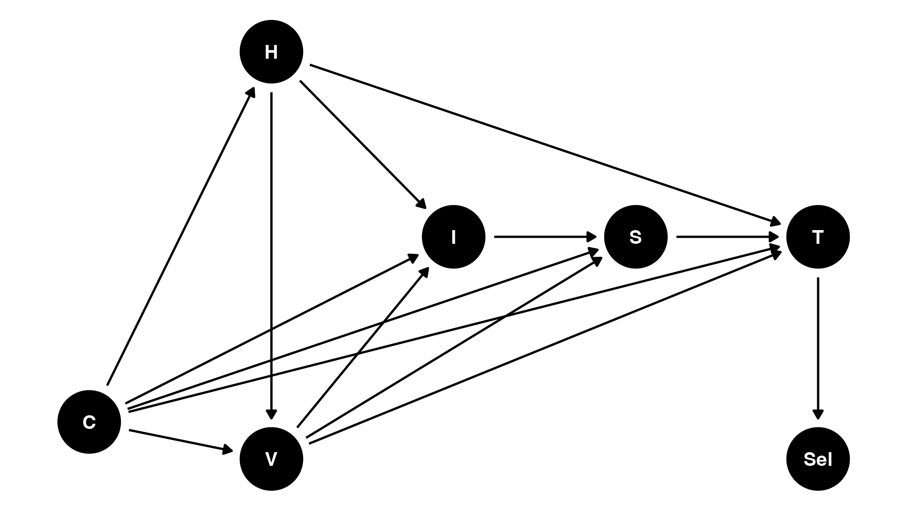


**Abbreviations**: $C$: Confounders; $H$: health and healthcare-seeking behaviors; $I$: Infection status; $Ix$: Measured infection status; $S$: COVID-19-like symptoms; $Sel$: selection into the study sample; $V$: COVID-19 vaccination status; $T$: SARS-CoV-2 diagnostic tests.

# Supplementary Tables

## **Table S1**: Data generation process for the DAG-guided simulation study.

| Variable | Data Generation Description |
| --- | --- |
| *C*: Confounders (continuous) | The node "confounders" ($C$) was generated as a continuous, normally distributed variable with a mean of 40 and a standard deviation of 4 to represent it as an age variable, covering an approximate age range of 20 to 60 years. |
| *H*: Health and healthcare-seeking behaviors (HSBs; binary: present vs. absent) | The node "HSBs" ($H$) was generated as a Bernoulli distributed variable, calibrated to achieve a prevalence of approximately 0.365, indicating that 36.5% of members would seek care if they experienced COVID-19-like symptoms.[^7^](#_ENREF_7) Moreover, we assumed that increasing age ($C$) would raise the probability of exhibiting HSBs ($H=1$) within the simulated dataset.[^8^](#_ENREF_8) |
| *V*: COVID-19 vaccination status (binary: vaccinated vs. unvaccinated) | The node "vaccination status" ($V$) was simulated as a Bernoulli distributed variable, calibrated to achieve a prevalence of ~ 54.6%, representing the proportion of fully vaccinated individuals in the US as of September 1, 2021.[^9^](#_ENREF_9)^,^[^10^](#_ENREF_10) Additionally, we assumed that both HSBs ($H=1$) (e.g., regular physician visits) and older age ($C$) were positively associated with vaccine uptake.[^11^](#_ENREF_11) |
| *I =* 1: Non-SARS CoV-2 infection (binary: present vs. absent), *I =* 2: SARS-CoV-2 infection (binary: present vs. absent), and $Ix=2$: Measured SARS-CoV-2 Infection (binary: test positive vs. test negative). | The node "infection" ($I$) was generated as two separate Bernoulli distributed variables: 1) " non-SARS-CoV-2 infection" ($I=1$), and 2) "SARS-CoV-2 infection" ($I=2$), targeting prevalences of ~10% and ~12.2%, respectively. The latter corresponds to the estimated cumulative prevalence of confirmed SARS-CoV-2 infections in the US by September 1st, 2021.[^12^](#_ENREF_12) Both levels, $I=1$ and $I=2$, were generated under the assumption that individuals exhibiting HSBs ($H=1$) also engage in preventive behaviors that reduce their odds of infection (e.g., hand-washing[^13^](#_ENREF_13)), and that age ($C$) would increase an individual's probability of infection.[^14^](#_ENREF_14) On the other hand, to adhere to the TND assumption that COVID-19 vaccination does not affect the probability of contracting other infections that may cause COVID-19-like symptoms,[^15^](#_ENREF_15) we assumed that COVID-19 vaccination influences only the probability of SARS-CoV-2 infection ($I=2)$ but not the probability of any other infection ($I=1$), with a COVID-19 vaccine effectiveness against SARS-CoV-2 infection of 90% (i.e., an odds ratio of 0.1). The prevalence of other infections was set at 10% to account for the fact that $I=1$ could include not only other infections but also other test-negative illnesses presenting with COVID-19-like symptoms, such as allergies.  The variable "measured SARS-CoV-2 infection" ($Ix=2$) was generated as a Bernoulli distributed variable, with success probabilities depending on the presence of symptoms ($S$) and the true SARS-CoV-2 infection status ($I=2$). For the sake of our discussion, we simulate an extreme scenario in which all symptomatic individuals are tested using a molecular test with a sensitivity of approximately 97.1%,[^16^](#_ENREF_16) as documented for some tests in symptomatic cases, while all asymptomatic individuals are tested with a rapid antigen test with a sensitivity of approximately 58.1%, as reported elsewhere.[^17^](#_ENREF_17) We assume a specificity of 98.9% for both types of tests (antigen- and molecular-based), in line with findings from other studies.[^16^](#_ENREF_16)^,^[^17^](#_ENREF_17) |
| *S*: COVID-19-like symptoms (binary: symptomatic vs. asymptomatic) | The node "symptoms" ($S$) was generated as a Bernoulli distributed variable, calibrated to a prevalence of asymptomatic infection of 35% among those with SARS-CoV-2 infection ($I=2$).[^18^](#_ENREF_18) Furthermore, we allowed only individuals with either any other infection or a SARS-CoV-2 infection $(I=1$ or $I=2$, respectively) to have symptoms. For both types of infection, we assumed that older individuals would be more likely to have symptoms;[^18^](#_ENREF_18) however, we also assumed that only individuals with a SARS-CoV-2 infection ($I=2$) would have a reduced probability of having symptoms if they had previously been vaccinated against COVID-19 ($V=1$).[^19-21^](#_ENREF_19) |
| *T*: SARS-CoV-2 diagnostic tests (binary: tested vs. not tested) | The node "testing" ($T$) was simulated as a Bernoulli distributed variable, calibrated to a prevalence of 27.7%. This percentage represents the proportion of individuals who reported having used a SARS-CoV-2 diagnostic test within the past 30 days in an online survey of U.S. adults conducted between August 23, 2021, and March 12, 2022.[^22^](#_ENREF_22)  We assumed that symptomatic individuals would be more likely to seek testing.[^23^](#_ENREF_23) Furthermore, to be consistent with the TND assumption that all symptomatic and tested individuals exhibit HSBs ($H=1$),[^24^](#_ENREF_24)^,^[^25^](#_ENREF_25) we further assumed that all symptomatic individuals ($S=1$) with HSBs ($H=1$) would be tested, whereas symptomatic individuals ($S=1$) without HSBs ($H=0$) would not be tested. In addition, we hypothesized that older individuals ($C$) would be more likely to voluntarily seek testing, whereas those who had been vaccinated against COVID-19 ($V=1$) might be less likely to voluntarily undergo testing or might be less likely to be subjected to mandatory testing.[^26-28^](#_ENREF_26) The following is a summary of several scenarios in which mandatory testing policies differed for vaccinated and unvaccinated individuals during the COVID-19 pandemic:   - In the United States, the Occupational Safety and Health Administration (OSHA) recommended periodic COVID-19 testing for employees who were not fully vaccinated (this policy did not extend to fully vaccinated employees).[^27^](#_ENREF_27) - The U.S. Centers for Disease Control and Prevention (CDC) advised that fully vaccinated individuals could travel internationally without a COVID-19 test prior to departure unless required by the destination country.[^26^](#_ENREF_26) - The Public Health Agency of Canada noted that asymptomatic screening of vaccinated individuals could be considered mainly under certain conditions, such as elevated community prevalence or outbreaks.[^28^](#_ENREF_28) The agency also summarized several international recommendations, including those from Germany, which exempted individuals who had recovered from COVID-19 and those who were fully vaccinated from testing requirements, and Austria, which exempted vaccinated individuals from mandatory testing. In addition, the United Kingdom offered rapid antigen diagnostic tests primarily to unvaccinated individuals, and the EU CDC proposed eliminating routine asymptomatic testing for vaccinated individuals. |

## **Table S2**: Bias in odds ratio (OR) estimates for symptomatic COVID-19 (${OR}_{COVID}$) and SARS-CoV-2 infection (${OR}_{infect}$) relative to their target parameters (${RR}_{COVID}$ and ${RR}_{infect}$, respectively) after individually varying the strength of one selected parameters (assuming perfect SARS-CoV-2 diagnostic tests).

| OR HV | OR VT | I2 Prevalence | OR HI | OR HT | OR IS | OR IVS | OR ST | T Prevalence | Bias Classical | Bias Alternative |
| --- | --- | --- | --- | --- | --- | --- | --- | --- | --- | --- |
| 1.5 | 0.92 | 0.122 | 0.9 | 1.3 | 10 | 0.52 | 6 | 0.277 | -0.007 | -0.022 |
| 2.5 | 0.92 | 0.122 | 0.9 | 1.3 | 10 | 0.52 | 6 | 0.277 | -0.009 | -0.004 |
| 3.5 | 0.92 | 0.122 | 0.9 | 1.3 | 10 | 0.52 | 6 | 0.277 | -0.011 | 0.014 |
| 4.5 | 0.92 | 0.122 | 0.9 | 1.3 | 10 | 0.52 | 6 | 0.277 | -0.011 | 0.032 |
| 5.5 | 0.92 | 0.122 | 0.9 | 1.3 | 10 | 0.52 | 6 | 0.277 | -0.012 | 0.05 |
| 6.5 | 0.92 | 0.122 | 0.9 | 1.3 | 10 | 0.52 | 6 | 0.277 | -0.013 | 0.068 |
| 7.5 | 0.92 | 0.122 | 0.9 | 1.3 | 10 | 0.52 | 6 | 0.277 | -0.013 | 0.085 |
| 8.5 | 0.92 | 0.122 | 0.9 | 1.3 | 10 | 0.52 | 6 | 0.277 | -0.013 | 0.102 |
| 9.5 | 0.92 | 0.122 | 0.9 | 1.3 | 10 | 0.52 | 6 | 0.277 | -0.014 | 0.119 |
| 10.5 | 0.92 | 0.122 | 0.9 | 1.3 | 10 | 0.52 | 6 | 0.277 | -0.014 | 0.135 |
| 1.81 | 0.05 | 0.122 | 0.9 | 1.3 | 10 | 0.52 | 6 | 0.277 | -0.008 | 0.205 |
| 1.81 | 0.15 | 0.122 | 0.9 | 1.3 | 10 | 0.52 | 6 | 0.277 | -0.008 | 0.098 |
| 1.81 | 0.25 | 0.122 | 0.9 | 1.3 | 10 | 0.52 | 6 | 0.277 | -0.008 | 0.057 |
| 1.81 | 0.35 | 0.122 | 0.9 | 1.3 | 10 | 0.52 | 6 | 0.277 | -0.008 | 0.034 |
| 1.81 | 0.45 | 0.122 | 0.9 | 1.3 | 10 | 0.52 | 6 | 0.277 | -0.008 | 0.019 |
| 1.81 | 0.55 | 0.122 | 0.9 | 1.3 | 10 | 0.52 | 6 | 0.277 | -0.008 | 0.008 |
| 1.81 | 0.65 | 0.122 | 0.9 | 1.3 | 10 | 0.52 | 6 | 0.277 | -0.008 | -0.001 |
| 1.81 | 0.75 | 0.122 | 0.9 | 1.3 | 10 | 0.52 | 6 | 0.277 | -0.008 | -0.008 |
| 1.81 | 0.85 | 0.122 | 0.9 | 1.3 | 10 | 0.52 | 6 | 0.277 | -0.008 | -0.013 |
| 1.81 | 0.95 | 0.122 | 0.9 | 1.3 | 10 | 0.52 | 6 | 0.277 | -0.008 | -0.018 |
| 1.81 | 0.92 | 0.05 | 0.9 | 1.3 | 10 | 0.52 | 6 | 0.277 | -0.003 | 0.008 |
| 1.81 | 0.92 | 0.1 | 0.9 | 1.3 | 10 | 0.52 | 6 | 0.277 | -0.006 | -0.007 |
| 1.81 | 0.92 | 0.15 | 0.9 | 1.3 | 10 | 0.52 | 6 | 0.277 | -0.009 | -0.025 |
| 1.81 | 0.92 | 0.2 | 0.9 | 1.3 | 10 | 0.52 | 6 | 0.277 | -0.013 | -0.044 |
| 1.81 | 0.92 | 0.25 | 0.9 | 1.3 | 10 | 0.52 | 6 | 0.277 | -0.017 | -0.066 |
| 1.81 | 0.92 | 0.3 | 0.9 | 1.3 | 10 | 0.52 | 6 | 0.277 | -0.02 | -0.088 |
| 1.81 | 0.92 | 0.35 | 0.9 | 1.3 | 10 | 0.52 | 6 | 0.277 | -0.023 | -0.114 |
| 1.81 | 0.92 | 0.4 | 0.9 | 1.3 | 10 | 0.52 | 6 | 0.277 | -0.026 | -0.144 |
| 1.81 | 0.92 | 0.45 | 0.9 | 1.3 | 10 | 0.52 | 6 | 0.277 | -0.027 | -0.175 |
| 1.81 | 0.92 | 0.5 | 0.9 | 1.3 | 10 | 0.52 | 6 | 0.277 | -0.028 | -0.203 |
| 1.81 | 0.92 | 0.122 | 0.05 | 1.3 | 10 | 0.52 | 6 | 0.277 | -0.001 | -0.017 |
| 1.81 | 0.92 | 0.122 | 0.15 | 1.3 | 10 | 0.52 | 6 | 0.277 | -0.001 | -0.018 |
| 1.81 | 0.92 | 0.122 | 0.25 | 1.3 | 10 | 0.52 | 6 | 0.277 | -0.002 | -0.017 |
| 1.81 | 0.92 | 0.122 | 0.35 | 1.3 | 10 | 0.52 | 6 | 0.277 | -0.003 | -0.017 |
| 1.81 | 0.92 | 0.122 | 0.45 | 1.3 | 10 | 0.52 | 6 | 0.277 | -0.005 | -0.016 |
| 1.81 | 0.92 | 0.122 | 0.55 | 1.3 | 10 | 0.52 | 6 | 0.277 | -0.005 | -0.016 |
| 1.81 | 0.92 | 0.122 | 0.65 | 1.3 | 10 | 0.52 | 6 | 0.277 | -0.006 | -0.016 |
| 1.81 | 0.92 | 0.122 | 0.75 | 1.3 | 10 | 0.52 | 6 | 0.277 | -0.007 | -0.016 |
| 1.81 | 0.92 | 0.122 | 0.85 | 1.3 | 10 | 0.52 | 6 | 0.277 | -0.007 | -0.016 |
| 1.81 | 0.92 | 0.122 | 0.95 | 1.3 | 10 | 0.52 | 6 | 0.277 | -0.008 | -0.016 |
| 1.81 | 0.92 | 0.122 | 0.9 | 1.5 | 10 | 0.52 | 6 | 0.277 | -0.008 | -0.017 |
| 1.81 | 0.92 | 0.122 | 0.9 | 2.5 | 10 | 0.52 | 6 | 0.277 | -0.008 | -0.019 |
| 1.81 | 0.92 | 0.122 | 0.9 | 3.5 | 10 | 0.52 | 6 | 0.277 | -0.008 | -0.02 |
| 1.81 | 0.92 | 0.122 | 0.9 | 4.5 | 10 | 0.52 | 6 | 0.277 | -0.008 | -0.021 |
| 1.81 | 0.92 | 0.122 | 0.9 | 5.5 | 10 | 0.52 | 6 | 0.277 | -0.008 | -0.022 |
| 1.81 | 0.92 | 0.122 | 0.9 | 6.5 | 10 | 0.52 | 6 | 0.277 | -0.008 | -0.022 |
| 1.81 | 0.92 | 0.122 | 0.9 | 7.5 | 10 | 0.52 | 6 | 0.277 | -0.008 | -0.022 |
| 1.81 | 0.92 | 0.122 | 0.9 | 8.5 | 10 | 0.52 | 6 | 0.277 | -0.008 | -0.023 |
| 1.81 | 0.92 | 0.122 | 0.9 | 9.5 | 10 | 0.52 | 6 | 0.277 | -0.008 | -0.023 |
| 1.81 | 0.92 | 0.122 | 0.9 | 10.5 | 10 | 0.52 | 6 | 0.277 | -0.008 | -0.023 |
| 1.81 | 0.92 | 0.122 | 0.9 | 1.3 | 1.5 | 0.52 | 6 | 0.277 | -0.01 | -0.022 |
| 1.81 | 0.92 | 0.122 | 0.9 | 1.3 | 2.5 | 0.52 | 6 | 0.277 | -0.009 | -0.02 |
| 1.81 | 0.92 | 0.122 | 0.9 | 1.3 | 3.5 | 0.52 | 6 | 0.277 | -0.009 | -0.019 |
| 1.81 | 0.92 | 0.122 | 0.9 | 1.3 | 4.5 | 0.52 | 6 | 0.277 | -0.009 | -0.018 |
| 1.81 | 0.92 | 0.122 | 0.9 | 1.3 | 5.5 | 0.52 | 6 | 0.277 | -0.008 | -0.018 |
| 1.81 | 0.92 | 0.122 | 0.9 | 1.3 | 6.5 | 0.52 | 6 | 0.277 | -0.008 | -0.017 |
| 1.81 | 0.92 | 0.122 | 0.9 | 1.3 | 7.5 | 0.52 | 6 | 0.277 | -0.008 | -0.017 |
| 1.81 | 0.92 | 0.122 | 0.9 | 1.3 | 8.5 | 0.52 | 6 | 0.277 | -0.008 | -0.017 |
| 1.81 | 0.92 | 0.122 | 0.9 | 1.3 | 9.5 | 0.52 | 6 | 0.277 | -0.008 | -0.016 |
| 1.81 | 0.92 | 0.122 | 0.9 | 1.3 | 10.5 | 0.52 | 6 | 0.277 | -0.008 | -0.016 |
| 1.81 | 0.92 | 0.122 | 0.9 | 1.3 | 10 | 0.05 | 6 | 0.277 | -0.016 | -0.032 |
| 1.81 | 0.92 | 0.122 | 0.9 | 1.3 | 10 | 0.15 | 6 | 0.277 | -0.012 | -0.024 |
| 1.81 | 0.92 | 0.122 | 0.9 | 1.3 | 10 | 0.25 | 6 | 0.277 | -0.01 | -0.02 |
| 1.81 | 0.92 | 0.122 | 0.9 | 1.3 | 10 | 0.35 | 6 | 0.277 | -0.009 | -0.018 |
| 1.81 | 0.92 | 0.122 | 0.9 | 1.3 | 10 | 0.45 | 6 | 0.277 | -0.008 | -0.017 |
| 1.81 | 0.92 | 0.122 | 0.9 | 1.3 | 10 | 0.55 | 6 | 0.277 | -0.008 | -0.016 |
| 1.81 | 0.92 | 0.122 | 0.9 | 1.3 | 10 | 0.65 | 6 | 0.277 | -0.007 | -0.015 |
| 1.81 | 0.92 | 0.122 | 0.9 | 1.3 | 10 | 0.75 | 6 | 0.277 | -0.007 | -0.015 |
| 1.81 | 0.92 | 0.122 | 0.9 | 1.3 | 10 | 0.85 | 6 | 0.277 | -0.007 | -0.014 |
| 1.81 | 0.92 | 0.122 | 0.9 | 1.3 | 10 | 0.95 | 6 | 0.277 | -0.007 | -0.014 |
| 1.81 | 0.92 | 0.122 | 0.9 | 1.3 | 10 | 0.52 | 1.5 | 0.277 | -0.008 | -0.016 |
| 1.81 | 0.92 | 0.122 | 0.9 | 1.3 | 10 | 0.52 | 2.5 | 0.277 | -0.008 | -0.016 |
| 1.81 | 0.92 | 0.122 | 0.9 | 1.3 | 10 | 0.52 | 3.5 | 0.277 | -0.008 | -0.016 |
| 1.81 | 0.92 | 0.122 | 0.9 | 1.3 | 10 | 0.52 | 4.5 | 0.277 | -0.008 | -0.016 |
| 1.81 | 0.92 | 0.122 | 0.9 | 1.3 | 10 | 0.52 | 5.5 | 0.277 | -0.008 | -0.016 |
| 1.81 | 0.92 | 0.122 | 0.9 | 1.3 | 10 | 0.52 | 6.5 | 0.277 | -0.008 | -0.016 |
| 1.81 | 0.92 | 0.122 | 0.9 | 1.3 | 10 | 0.52 | 7.5 | 0.277 | -0.008 | -0.016 |
| 1.81 | 0.92 | 0.122 | 0.9 | 1.3 | 10 | 0.52 | 8.5 | 0.277 | -0.008 | -0.016 |
| 1.81 | 0.92 | 0.122 | 0.9 | 1.3 | 10 | 0.52 | 9.5 | 0.277 | -0.008 | -0.016 |
| 1.81 | 0.92 | 0.122 | 0.9 | 1.3 | 10 | 0.52 | 10.5 | 0.277 | -0.008 | -0.016 |
| 1.81 | 0.92 | 0.122 | 0.9 | 1.3 | 10 | 0.52 | 6 | 0.25 | -0.008 | -0.017 |
| 1.81 | 0.92 | 0.122 | 0.9 | 1.3 | 10 | 0.52 | 6 | 0.3 | -0.008 | -0.015 |
| 1.81 | 0.92 | 0.122 | 0.9 | 1.3 | 10 | 0.52 | 6 | 0.35 | -0.008 | -0.014 |
| 1.81 | 0.92 | 0.122 | 0.9 | 1.3 | 10 | 0.52 | 6 | 0.4 | -0.008 | -0.012 |
| 1.81 | 0.92 | 0.122 | 0.9 | 1.3 | 10 | 0.52 | 6 | 0.45 | -0.008 | -0.01 |
| 1.81 | 0.92 | 0.122 | 0.9 | 1.3 | 10 | 0.52 | 6 | 0.5 | -0.008 | -0.008 |
| 1.81 | 0.92 | 0.122 | 0.9 | 1.3 | 10 | 0.52 | 6 | 0.55 | -0.008 | -0.007 |
| 1.81 | 0.92 | 0.122 | 0.9 | 1.3 | 10 | 0.52 | 6 | 0.6 | -0.008 | -0.005 |
| 1.81 | 0.92 | 0.122 | 0.9 | 1.3 | 10 | 0.52 | 6 | 0.65 | -0.008 | -0.003 |
| 1.81 | 0.92 | 0.122 | 0.9 | 1.3 | 10 | 0.52 | 6 | 0.7 | -0.008 | -0.001 |

Bias formula = $exp(mean(\log\left( \hat{OR} \right)))- RR$.

**Abbreviations**: $H$: health and healthcare-seeking behaviors; $I$: Infection status; $I=2$: SARS-CoV-2 infection status; OR, odds ratio; RR, risk ratio; $T$: SARS-CoV-2 diagnostic tests; TND, test-negative design; $V$: COVID-19 vaccination status.

**Note:** Cells highlighted in yellow indicate the selected value for the parameter being varied.

## **Table S3**: Bias in odds ratio (OR) estimates for symptomatic COVID-19 (${OR}_{COVID}$) and SARS-CoV-2 infection (${OR}_{infect}$) relative to their target parameters (${RR}_{COVID}$ and ${RR}_{infect}$, respectively) after simultaneously varying the strength of two or three selected parameters (assuming perfect SARS-CoV-2 diagnostic tests).

| OR HV | OR VT | I2 Prevalence | Bias Classical | Bias Alternative |
| --- | --- | --- | --- | --- |
| 1.5 | 0.95 | 0.122 | -0.007 | -0.023 |
| 2.5 | 0.85 | 0.122 | -0.009 | 0.000 |
| 3.5 | 0.75 | 0.122 | -0.011 | 0.026 |
| 4.5 | 0.65 | 0.122 | -0.011 | 0.056 |
| 5.5 | 0.55 | 0.122 | -0.012 | 0.091 |
| 6.5 | 0.45 | 0.122 | -0.013 | 0.135 |
| 7.5 | 0.35 | 0.122 | -0.013 | 0.192 |
| 8.5 | 0.25 | 0.122 | -0.013 | 0.272 |
| 9.5 | 0.15 | 0.122 | -0.014 | 0.403 |
| 10.5 | 0.05 | 0.122 | -0.014 | 0.699 |
| 1.5 | 0.92 | 0.5 | -0.025 | -0.209 |
| 2.5 | 0.92 | 0.45 | -0.032 | -0.163 |
| 3.5 | 0.92 | 0.4 | -0.034 | -0.114 |
| 4.5 | 0.92 | 0.35 | -0.032 | -0.067 |
| 5.5 | 0.92 | 0.3 | -0.029 | -0.024 |
| 6.5 | 0.92 | 0.25 | -0.025 | 0.016 |
| 7.5 | 0.92 | 0.2 | -0.020 | 0.055 |
| 8.5 | 0.92 | 0.15 | -0.016 | 0.092 |
| 9.5 | 0.92 | 0.1 | -0.011 | 0.129 |
| 10.5 | 0.92 | 0.05 | -0.006 | 0.164 |
| 1.81 | 0.95 | 0.5 | -0.028 | -0.205 |
| 1.81 | 0.85 | 0.45 | -0.027 | -0.170 |
| 1.81 | 0.75 | 0.4 | -0.026 | -0.131 |
| 1.81 | 0.65 | 0.35 | -0.023 | -0.094 |
| 1.81 | 0.55 | 0.3 | -0.020 | -0.060 |
| 1.81 | 0.45 | 0.25 | -0.017 | -0.026 |
| 1.81 | 0.35 | 0.2 | -0.013 | 0.010 |
| 1.81 | 0.25 | 0.15 | -0.009 | 0.050 |
| 1.81 | 0.15 | 0.1 | -0.006 | 0.105 |
| 1.81 | 0.05 | 0.05 | -0.003 | 0.218 |
| 1.5 | 0.95 | 0.5 | -0.025 | -0.211 |
| 2.5 | 0.85 | 0.45 | -0.032 | -0.157 |
| 3.5 | 0.75 | 0.4 | -0.034 | -0.099 |
| 4.5 | 0.65 | 0.35 | -0.032 | -0.039 |
| 5.5 | 0.55 | 0.3 | -0.029 | 0.022 |
| 6.5 | 0.45 | 0.25 | -0.025 | 0.087 |
| 7.5 | 0.35 | 0.2 | -0.020 | 0.164 |
| 8.5 | 0.25 | 0.15 | -0.016 | 0.263 |
| 9.5 | 0.15 | 0.1 | -0.011 | 0.412 |
| 10.5 | 0.05 | 0.05 | -0.006 | 0.728 |

Bias formula = $exp(mean(\log\left( \hat{OR} \right)))- RR$.

**Abbreviations**: $H$: health and healthcare-seeking behaviors; $I$: Infection status; $I=2$: SARS-CoV-2 infection status; OR, odds ratio; RR, risk ratio; $T$: SARS-CoV-2 diagnostic tests; TND, test-negative design; $V$: COVID-19 vaccination status.

**Note:** Cells highlighted in yellow indicate the selected value for the parameter being varied.

## **Table S4**: Bias in odds ratio (OR) estimates for **measured** symptomatic COVID-19 (${OR}_{COVID}$) and **measured** SARS-CoV-2 infection (${OR}_{infect}$) relative to their target parameters (${RR}_{COVID}$ and ${RR}_{infect}$, respectively) after individually varying the strength of selected parameters (assuming symptomatic individuals are tested exclusively with NAATs and asymptomatic individuals are tested exclusively with antigen tests).

| OR HV | OR VT | I2 Prevalence | OR HI | OR HT | OR IS | OR IVS | OR ST | T Prevalence | Bias Classical | Bias Alternative |
| --- | --- | --- | --- | --- | --- | --- | --- | --- | --- | --- |
| 1.5 | 0.92 | 0.122 | 0.9 | 1.3 | 10 | 0.52 | 6 | 0.277 | 0.006 | -0.002 |
| 2.5 | 0.92 | 0.122 | 0.9 | 1.3 | 10 | 0.52 | 6 | 0.277 | 0.003 | 0.018 |
| 3.5 | 0.92 | 0.122 | 0.9 | 1.3 | 10 | 0.52 | 6 | 0.277 | 0.002 | 0.039 |
| 4.5 | 0.92 | 0.122 | 0.9 | 1.3 | 10 | 0.52 | 6 | 0.277 | 0.001 | 0.059 |
| 5.5 | 0.92 | 0.122 | 0.9 | 1.3 | 10 | 0.52 | 6 | 0.277 | 0 | 0.079 |
| 6.5 | 0.92 | 0.122 | 0.9 | 1.3 | 10 | 0.52 | 6 | 0.277 | 0 | 0.098 |
| 7.5 | 0.92 | 0.122 | 0.9 | 1.3 | 10 | 0.52 | 6 | 0.277 | 0 | 0.118 |
| 8.5 | 0.92 | 0.122 | 0.9 | 1.3 | 10 | 0.52 | 6 | 0.277 | -0.001 | 0.137 |
| 9.5 | 0.92 | 0.122 | 0.9 | 1.3 | 10 | 0.52 | 6 | 0.277 | -0.001 | 0.156 |
| 10.5 | 0.92 | 0.122 | 0.9 | 1.3 | 10 | 0.52 | 6 | 0.277 | -0.001 | 0.175 |
| 1.81 | 0.05 | 0.122 | 0.9 | 1.3 | 10 | 0.52 | 6 | 0.277 | 0.005 | 0.226 |
| 1.81 | 0.15 | 0.122 | 0.9 | 1.3 | 10 | 0.52 | 6 | 0.277 | 0.005 | 0.12 |
| 1.81 | 0.25 | 0.122 | 0.9 | 1.3 | 10 | 0.52 | 6 | 0.277 | 0.005 | 0.079 |
| 1.81 | 0.35 | 0.122 | 0.9 | 1.3 | 10 | 0.52 | 6 | 0.277 | 0.005 | 0.055 |
| 1.81 | 0.45 | 0.122 | 0.9 | 1.3 | 10 | 0.52 | 6 | 0.277 | 0.005 | 0.04 |
| 1.81 | 0.55 | 0.122 | 0.9 | 1.3 | 10 | 0.52 | 6 | 0.277 | 0.005 | 0.028 |
| 1.81 | 0.65 | 0.122 | 0.9 | 1.3 | 10 | 0.52 | 6 | 0.277 | 0.005 | 0.02 |
| 1.81 | 0.75 | 0.122 | 0.9 | 1.3 | 10 | 0.52 | 6 | 0.277 | 0.005 | 0.013 |
| 1.81 | 0.85 | 0.122 | 0.9 | 1.3 | 10 | 0.52 | 6 | 0.277 | 0.005 | 0.007 |
| 1.81 | 0.95 | 0.122 | 0.9 | 1.3 | 10 | 0.52 | 6 | 0.277 | 0.005 | 0.003 |
| 1.81 | 0.92 | 0.05 | 0.9 | 1.3 | 10 | 0.52 | 6 | 0.277 | 0.01 | 0.06 |
| 1.81 | 0.92 | 0.1 | 0.9 | 1.3 | 10 | 0.52 | 6 | 0.277 | 0.006 | 0.019 |
| 1.81 | 0.92 | 0.15 | 0.9 | 1.3 | 10 | 0.52 | 6 | 0.277 | 0.005 | -0.008 |
| 1.81 | 0.92 | 0.2 | 0.9 | 1.3 | 10 | 0.52 | 6 | 0.277 | 0.005 | -0.03 |
| 1.81 | 0.92 | 0.25 | 0.9 | 1.3 | 10 | 0.52 | 6 | 0.277 | 0.005 | -0.053 |
| 1.81 | 0.92 | 0.3 | 0.9 | 1.3 | 10 | 0.52 | 6 | 0.277 | 0.006 | -0.075 |
| 1.81 | 0.92 | 0.35 | 0.9 | 1.3 | 10 | 0.52 | 6 | 0.277 | 0.008 | -0.099 |
| 1.81 | 0.92 | 0.4 | 0.9 | 1.3 | 10 | 0.52 | 6 | 0.277 | 0.01 | -0.126 |
| 1.81 | 0.92 | 0.45 | 0.9 | 1.3 | 10 | 0.52 | 6 | 0.277 | 0.013 | -0.154 |
| 1.81 | 0.92 | 0.5 | 0.9 | 1.3 | 10 | 0.52 | 6 | 0.277 | 0.015 | -0.179 |
| 1.81 | 0.92 | 0.122 | 0.05 | 1.3 | 10 | 0.52 | 6 | 0.277 | 0.012 | 0.152 |
| 1.81 | 0.92 | 0.122 | 0.15 | 1.3 | 10 | 0.52 | 6 | 0.277 | 0.011 | 0.055 |
| 1.81 | 0.92 | 0.122 | 0.25 | 1.3 | 10 | 0.52 | 6 | 0.277 | 0.01 | 0.031 |
| 1.81 | 0.92 | 0.122 | 0.35 | 1.3 | 10 | 0.52 | 6 | 0.277 | 0.009 | 0.02 |
| 1.81 | 0.92 | 0.122 | 0.45 | 1.3 | 10 | 0.52 | 6 | 0.277 | 0.008 | 0.014 |
| 1.81 | 0.92 | 0.122 | 0.55 | 1.3 | 10 | 0.52 | 6 | 0.277 | 0.007 | 0.01 |
| 1.81 | 0.92 | 0.122 | 0.65 | 1.3 | 10 | 0.52 | 6 | 0.277 | 0.007 | 0.008 |
| 1.81 | 0.92 | 0.122 | 0.75 | 1.3 | 10 | 0.52 | 6 | 0.277 | 0.006 | 0.006 |
| 1.81 | 0.92 | 0.122 | 0.85 | 1.3 | 10 | 0.52 | 6 | 0.277 | 0.005 | 0.005 |
| 1.81 | 0.92 | 0.122 | 0.95 | 1.3 | 10 | 0.52 | 6 | 0.277 | 0.005 | 0.004 |
| 1.81 | 0.92 | 0.122 | 0.9 | 1.5 | 10 | 0.52 | 6 | 0.277 | 0.005 | 0.004 |
| 1.81 | 0.92 | 0.122 | 0.9 | 2.5 | 10 | 0.52 | 6 | 0.277 | 0.005 | 0.003 |
| 1.81 | 0.92 | 0.122 | 0.9 | 3.5 | 10 | 0.52 | 6 | 0.277 | 0.005 | 0.002 |
| 1.81 | 0.92 | 0.122 | 0.9 | 4.5 | 10 | 0.52 | 6 | 0.277 | 0.005 | 0.002 |
| 1.81 | 0.92 | 0.122 | 0.9 | 5.5 | 10 | 0.52 | 6 | 0.277 | 0.005 | 0.002 |
| 1.81 | 0.92 | 0.122 | 0.9 | 6.5 | 10 | 0.52 | 6 | 0.277 | 0.005 | 0.002 |
| 1.81 | 0.92 | 0.122 | 0.9 | 7.5 | 10 | 0.52 | 6 | 0.277 | 0.005 | 0.002 |
| 1.81 | 0.92 | 0.122 | 0.9 | 8.5 | 10 | 0.52 | 6 | 0.277 | 0.005 | 0.002 |
| 1.81 | 0.92 | 0.122 | 0.9 | 9.5 | 10 | 0.52 | 6 | 0.277 | 0.005 | 0.002 |
| 1.81 | 0.92 | 0.122 | 0.9 | 10.5 | 10 | 0.52 | 6 | 0.277 | 0.005 | 0.002 |
| 1.81 | 0.92 | 0.122 | 0.9 | 1.3 | 1.5 | 0.52 | 6 | 0.277 | 0.003 | 0.001 |
| 1.81 | 0.92 | 0.122 | 0.9 | 1.3 | 2.5 | 0.52 | 6 | 0.277 | 0.003 | 0.002 |
| 1.81 | 0.92 | 0.122 | 0.9 | 1.3 | 3.5 | 0.52 | 6 | 0.277 | 0.004 | 0.002 |
| 1.81 | 0.92 | 0.122 | 0.9 | 1.3 | 4.5 | 0.52 | 6 | 0.277 | 0.004 | 0.003 |
| 1.81 | 0.92 | 0.122 | 0.9 | 1.3 | 5.5 | 0.52 | 6 | 0.277 | 0.004 | 0.003 |
| 1.81 | 0.92 | 0.122 | 0.9 | 1.3 | 6.5 | 0.52 | 6 | 0.277 | 0.005 | 0.003 |
| 1.81 | 0.92 | 0.122 | 0.9 | 1.3 | 7.5 | 0.52 | 6 | 0.277 | 0.005 | 0.004 |
| 1.81 | 0.92 | 0.122 | 0.9 | 1.3 | 8.5 | 0.52 | 6 | 0.277 | 0.005 | 0.004 |
| 1.81 | 0.92 | 0.122 | 0.9 | 1.3 | 9.5 | 0.52 | 6 | 0.277 | 0.005 | 0.004 |
| 1.81 | 0.92 | 0.122 | 0.9 | 1.3 | 10.5 | 0.52 | 6 | 0.277 | 0.005 | 0.004 |
| 1.81 | 0.92 | 0.122 | 0.9 | 1.3 | 10 | 0.05 | 6 | 0.277 | -0.005 | -0.017 |
| 1.81 | 0.92 | 0.122 | 0.9 | 1.3 | 10 | 0.15 | 6 | 0.277 | 0.001 | -0.005 |
| 1.81 | 0.92 | 0.122 | 0.9 | 1.3 | 10 | 0.25 | 6 | 0.277 | 0.003 | -0.001 |
| 1.81 | 0.92 | 0.122 | 0.9 | 1.3 | 10 | 0.35 | 6 | 0.277 | 0.004 | 0.001 |
| 1.81 | 0.92 | 0.122 | 0.9 | 1.3 | 10 | 0.45 | 6 | 0.277 | 0.005 | 0.003 |
| 1.81 | 0.92 | 0.122 | 0.9 | 1.3 | 10 | 0.55 | 6 | 0.277 | 0.005 | 0.004 |
| 1.81 | 0.92 | 0.122 | 0.9 | 1.3 | 10 | 0.65 | 6 | 0.277 | 0.006 | 0.005 |
| 1.81 | 0.92 | 0.122 | 0.9 | 1.3 | 10 | 0.75 | 6 | 0.277 | 0.006 | 0.006 |
| 1.81 | 0.92 | 0.122 | 0.9 | 1.3 | 10 | 0.85 | 6 | 0.277 | 0.006 | 0.007 |
| 1.81 | 0.92 | 0.122 | 0.9 | 1.3 | 10 | 0.95 | 6 | 0.277 | 0.006 | 0.007 |
| 1.81 | 0.92 | 0.122 | 0.9 | 1.3 | 10 | 0.52 | 1.5 | 0.277 | 0.005 | 0.004 |
| 1.81 | 0.92 | 0.122 | 0.9 | 1.3 | 10 | 0.52 | 2.5 | 0.277 | 0.005 | 0.004 |
| 1.81 | 0.92 | 0.122 | 0.9 | 1.3 | 10 | 0.52 | 3.5 | 0.277 | 0.005 | 0.004 |
| 1.81 | 0.92 | 0.122 | 0.9 | 1.3 | 10 | 0.52 | 4.5 | 0.277 | 0.005 | 0.004 |
| 1.81 | 0.92 | 0.122 | 0.9 | 1.3 | 10 | 0.52 | 5.5 | 0.277 | 0.005 | 0.004 |
| 1.81 | 0.92 | 0.122 | 0.9 | 1.3 | 10 | 0.52 | 6.5 | 0.277 | 0.005 | 0.004 |
| 1.81 | 0.92 | 0.122 | 0.9 | 1.3 | 10 | 0.52 | 7.5 | 0.277 | 0.005 | 0.004 |
| 1.81 | 0.92 | 0.122 | 0.9 | 1.3 | 10 | 0.52 | 8.5 | 0.277 | 0.005 | 0.004 |
| 1.81 | 0.92 | 0.122 | 0.9 | 1.3 | 10 | 0.52 | 9.5 | 0.277 | 0.005 | 0.004 |
| 1.81 | 0.92 | 0.122 | 0.9 | 1.3 | 10 | 0.52 | 10.5 | 0.277 | 0.005 | 0.004 |
| 1.81 | 0.92 | 0.122 | 0.9 | 1.3 | 10 | 0.52 | 6 | 0.25 | 0.005 | 0.001 |
| 1.81 | 0.92 | 0.122 | 0.9 | 1.3 | 10 | 0.52 | 6 | 0.3 | 0.005 | 0.007 |
| 1.81 | 0.92 | 0.122 | 0.9 | 1.3 | 10 | 0.52 | 6 | 0.35 | 0.005 | 0.013 |
| 1.81 | 0.92 | 0.122 | 0.9 | 1.3 | 10 | 0.52 | 6 | 0.4 | 0.005 | 0.02 |
| 1.81 | 0.92 | 0.122 | 0.9 | 1.3 | 10 | 0.52 | 6 | 0.45 | 0.005 | 0.026 |
| 1.81 | 0.92 | 0.122 | 0.9 | 1.3 | 10 | 0.52 | 6 | 0.5 | 0.005 | 0.032 |
| 1.81 | 0.92 | 0.122 | 0.9 | 1.3 | 10 | 0.52 | 6 | 0.55 | 0.005 | 0.037 |
| 1.81 | 0.92 | 0.122 | 0.9 | 1.3 | 10 | 0.52 | 6 | 0.6 | 0.005 | 0.042 |
| 1.81 | 0.92 | 0.122 | 0.9 | 1.3 | 10 | 0.52 | 6 | 0.65 | 0.005 | 0.045 |
| 1.81 | 0.92 | 0.122 | 0.9 | 1.3 | 10 | 0.52 | 6 | 0.7 | 0.005 | 0.049 |

Bias formula = $exp(mean(\log\left( \hat{OR} \right)))- RR$.

**Abbreviations**: $H$: health and healthcare-seeking behaviors; $I$: Infection status; $I=2$: SARS-CoV-2 infection status; OR, odds ratio; RR, risk ratio; $T$: SARS-CoV-2 diagnostic tests; TND, test-negative design; $V$: COVID-19 vaccination status.

**Note:** Cells highlighted in yellow indicate the selected value for the parameter being varied.

## **Table S5**: Bias in odds ratio (OR) estimates for **measured** symptomatic COVID-19 (${OR}_{COVID}$) and **measured** SARS-CoV-2 infection (${OR}_{infect}$) relative to their target parameters (${RR}_{COVID}$ and ${RR}_{infect}$, respectively) after individually varying the strength of two or three selected parameters (assuming symptomatic individuals are tested exclusively with NAATs and asymptomatic individuals are tested exclusively with antigen tests).

| OR HV | OR VT | I2 Prevalence | Bias Classical | Bias Alternative |
| --- | --- | --- | --- | --- |
| 1.5 | 0.95 | 0.122 | 0.006 | -0.004 |
| 2.5 | 0.85 | 0.122 | 0.003 | 0.022 |
| 3.5 | 0.75 | 0.122 | 0.002 | 0.050 |
| 4.5 | 0.65 | 0.122 | 0.001 | 0.083 |
| 5.5 | 0.55 | 0.122 | 0.000 | 0.120 |
| 6.5 | 0.45 | 0.122 | 0.000 | 0.166 |
| 7.5 | 0.35 | 0.122 | 0.000 | 0.225 |
| 8.5 | 0.25 | 0.122 | -0.001 | 0.307 |
| 9.5 | 0.15 | 0.122 | -0.001 | 0.439 |
| 10.5 | 0.05 | 0.122 | -0.001 | 0.734 |
| 1.5 | 0.92 | 0.5 | 0.018 | -0.186 |
| 2.5 | 0.92 | 0.45 | 0.009 | -0.140 |
| 3.5 | 0.92 | 0.4 | 0.003 | -0.093 |
| 4.5 | 0.92 | 0.35 | -0.001 | -0.047 |
| 5.5 | 0.92 | 0.3 | -0.003 | -0.004 |
| 6.5 | 0.92 | 0.25 | -0.003 | 0.037 |
| 7.5 | 0.92 | 0.2 | -0.003 | 0.079 |
| 8.5 | 0.92 | 0.15 | -0.002 | 0.122 |
| 9.5 | 0.92 | 0.1 | 0.001 | 0.175 |
| 10.5 | 0.92 | 0.05 | 0.007 | 0.255 |
| 1.81 | 0.95 | 0.5 | 0.015 | -0.181 |
| 1.81 | 0.85 | 0.45 | 0.013 | -0.149 |
| 1.81 | 0.75 | 0.4 | 0.010 | -0.113 |
| 1.81 | 0.65 | 0.35 | 0.008 | -0.078 |
| 1.81 | 0.55 | 0.3 | 0.006 | -0.044 |
| 1.81 | 0.45 | 0.25 | 0.005 | -0.011 |
| 1.81 | 0.35 | 0.2 | 0.005 | 0.025 |
| 1.81 | 0.25 | 0.15 | 0.005 | 0.068 |
| 1.81 | 0.15 | 0.1 | 0.006 | 0.131 |
| 1.81 | 0.05 | 0.05 | 0.010 | 0.265 |
| 1.5 | 0.95 | 0.5 | 0.018 | -0.188 |
| 2.5 | 0.85 | 0.45 | 0.009 | -0.134 |
| 3.5 | 0.75 | 0.4 | 0.003 | -0.076 |
| 4.5 | 0.65 | 0.35 | -0.001 | -0.017 |
| 5.5 | 0.55 | 0.3 | -0.003 | 0.044 |
| 6.5 | 0.45 | 0.25 | -0.003 | 0.111 |
| 7.5 | 0.35 | 0.2 | -0.003 | 0.191 |
| 8.5 | 0.25 | 0.15 | -0.002 | 0.295 |
| 9.5 | 0.15 | 0.1 | 0.001 | 0.452 |
| 10.5 | 0.05 | 0.05 | 0.007 | 0.766 |

Bias formula = $exp(mean(\log\left( \hat{OR} \right)))- RR$.

**Abbreviations**: $H$: health and healthcare-seeking behaviors; $I$: Infection status; $I=2$: SARS-CoV-2 infection status; OR, odds ratio; RR, risk ratio; $T$: SARS-CoV-2 diagnostic tests; TND, test-negative design; $V$: COVID-19 vaccination status.

**Note:** Cells highlighted in yellow indicate the selected value for the parameter being varied.

# Supplementary Figures

## **Figure S1**: Causal directed acyclic graphs for "alternative" test-negative design studies of COVID-19 vaccine effectiveness - highlighting the remaining open paths between vaccination status $(V)$ and the outcome infection ($I$) after restricting the study sample to tested individuals ($T=1$).


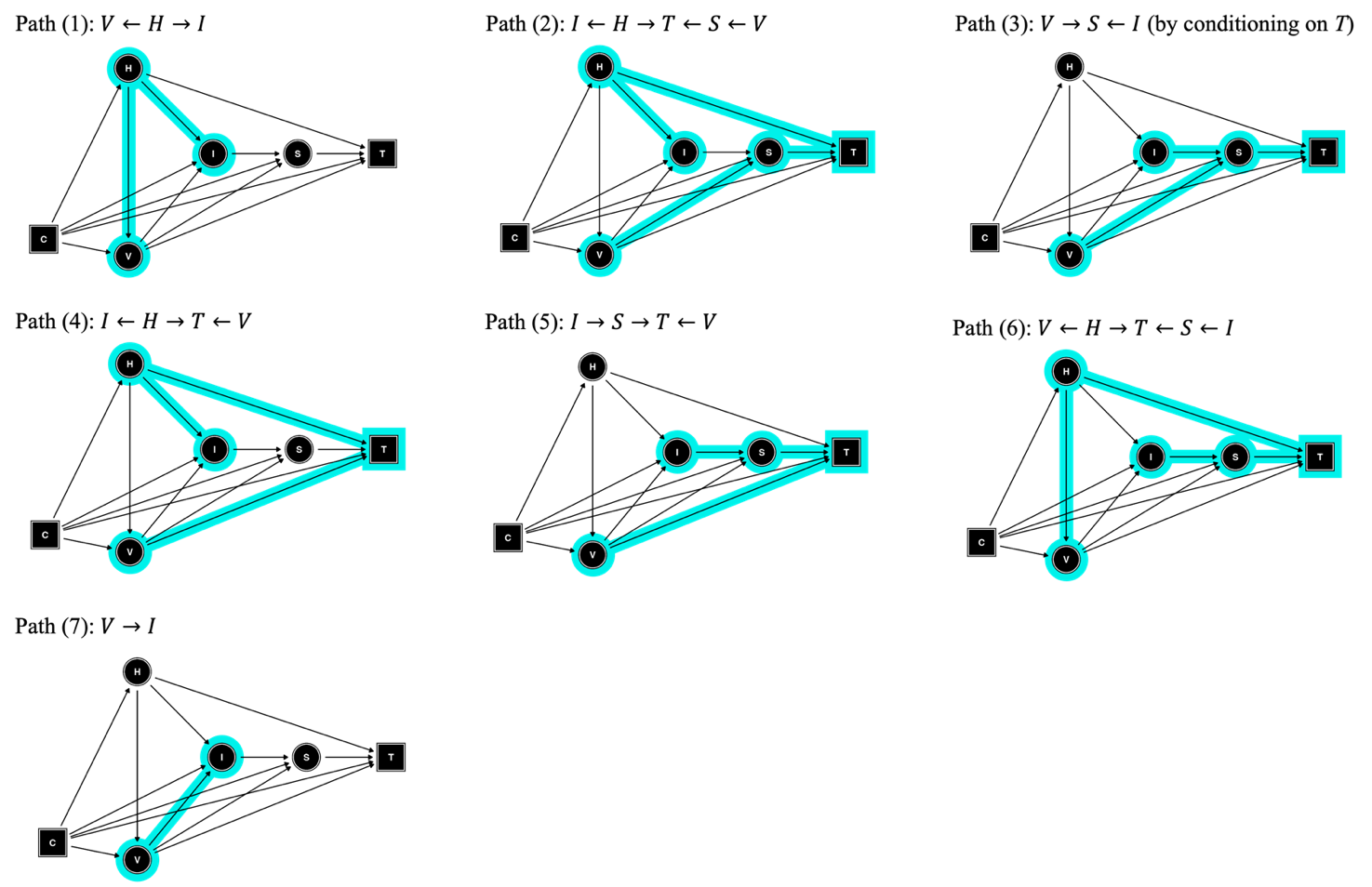


**Abbreviations**: $C$: Confounders; $H$: health and healthcare-seeking behaviors; $I$: Infection status; $Ix$: Measured infection status; $S$: COVID-19-like symptoms; $T$: SARS-CoV-2 diagnostic tests; $V$: COVID-19 vaccination status.

**Notes**:

- The square nodes indicate that the variable is controlled by either the study design or the analysis, while unconditioned variables are represented as circles.
- Biasing path leading to uncontrolled confounding by healthcare-seeking behaviors: $V\leftarrow H\to I$ (path 1).
- Biasing paths leading to collider stratification bias: $I\leftarrow H\to T\leftarrow S\leftarrow V$ (path 2); $V\to S\leftarrow I$(path 3); $I\leftarrow H\to T\leftarrow V$ (path 4); $I\to S\to T\leftarrow V$ (path 5); $V\leftarrow H\to T\leftarrow S\leftarrow I$ (path 6).

# Appendix References

1. Hernán MA RJ. Chapter 6. Graphical representation of causal effects. Causal Inference: What If: Chapman & Hall/CRC; 2020: 69-82.

2. Westreich D. Epidemiology by Design: A Causal Approach to the Health Sciences: Oxford University Press; 2019.

3. Lash TL, VanderWeele TJ, Haneuse S, Rothman KJ. Modern Epidemiology. 4th_Edition ed: Lippincott Williams & Wilkins; 2021. p. 1-1174.

4. Infante-Rivard C, Cusson A. Reflection on modern methods: selection bias-a review of recent developments. *Int J Epidemiol* 2018; **47**(5): 1714-22.

5. Ciocanea-Teodorescu I, Nason M, Sjolander A, Gabriel EE. Adjustment for Disease Severity in the Test-Negative Study Design. *Am J Epidemiol* 2021; **190**(9): 1882-9.

6. Didelez V, Kreiner S, Keiding N. Graphical Models for Inference Under Outcome-Dependent Sampling. *Statistical Science* 2010; **25**(3): 368-87, 20.

7. Yang J, Gong H, Chen X, et al. Health-seeking behaviors of patients with acute respiratory infections during the outbreak of novel coronavirus disease 2019 in Wuhan, China. *Influenza Other Respir Viruses* 2021; **15**(2): 188-94.

8. Chilot D, Shitu K, Gela YY, et al. Factors associated with healthcare-seeking behavior for symptomatic acute respiratory infection among children in East Africa: a cross-sectional study. *BMC Pediatr* 2022; **22**(1): 662.

9. U.S. Census Bureau Population and Housing Unit Estimates for the United States. <https://www.census.gov/programs-surveys/popest.html>. Published September 1, 2021. Updated May 6, 2024. Accessed April 10, 2023.

10. Mathieu E, Ritchie H, Ortiz-Ospina E, et al. A global database of COVID-19 vaccinations. *Nat Hum Behav* 2021; **5**(7): 947-53.

11. Burch AE, Lee E, Shackelford P, Schmidt P, Bolin P. Willingness to Vaccinate Against COVID-19: Predictors of Vaccine Uptake Among Adults in the US. *J Prev (2022)* 2022; **43**(1): 83-93.

12. World Health Organization. United States of America: WHO Coronavirus (COVID-19) Dashboard. <https://covid19.who.int/region/amro/country/us>. Published September 1, 2021. Updated May 18, 2024. Accessed April 10, 2023.

13. Wong VW, Cowling BJ, Aiello AE. Hand hygiene and risk of influenza virus infections in the community: a systematic review and meta-analysis. *Epidemiol Infect* 2014; **142**(5): 922-32.

14. Dai CL, Kornilov SA, Roper RT, et al. Characteristics and Factors Associated With Coronavirus Disease 2019 Infection, Hospitalization, and Mortality Across Race and Ethnicity. *Clin Infect Dis* 2021; **73**(12): 2193-204.

15. Schnitzer ME. Estimands and Estimation of COVID-19 Vaccine Effectiveness Under the Test-Negative Design: Connections to Causal Inference. *Epidemiology* 2022; **33**(3): 325-33.

16. Hohl CM, Hau JP, Vaillancourt S, et al. Sensitivity and Diagnostic Yield of the First SARS-CoV-2 Nucleic Acid Amplification Test Performed for Patients Presenting to the Hospital. *JAMA Netw Open* 2022; **5**(10): e2236288.

17. Dinnes J, Deeks JJ, Adriano A, et al. Rapid, point-of-care antigen and molecular-based tests for diagnosis of SARS-CoV-2 infection. *Cochrane Database Syst Rev* 2020; **8**(8): CD013705.

18. Sah P, Fitzpatrick MC, Zimmer CF, et al. Asymptomatic SARS-CoV-2 infection: A systematic review and meta-analysis. *Proc Natl Acad Sci U S A* 2021; **118**(34).

19. Antonelli M, Penfold RS, Merino J, et al. Risk factors and disease profile of post-vaccination SARS-CoV-2 infection in UK users of the COVID Symptom Study app: a prospective, community-based, nested, case-control study. *Lancet Infect Dis* 2022; **22**(1): 43-55.

20. Antonelli M, Penfold RS, Canas LDS, et al. SARS-CoV-2 infection following booster vaccination: illness and symptom profile in a prospective, observational community-based case-control study. *J Infect* 2023.

21. Grana C, Ghosn L, Evrenoglou T, et al. Efficacy and safety of COVID-19 vaccines. *Cochrane Database Syst Rev* 2022; **12**(12): CD015477.

22. Rader B, Gertz A, Iuliano AD, et al. Use of At-Home COVID-19 Tests - United States, August 23, 2021-March 12, 2022. *MMWR Morb Mortal Wkly Rep* 2022; **71**(13): 489-94.

23. Siegler AJ, Hall E, Luisi N, et al. Willingness to Seek Diagnostic Testing for SARS-CoV-2 With Home, Drive-through, and Clinic-Based Specimen Collection Locations. *Open Forum Infect Dis* 2020; **7**(7): ofaa269.

24. Jackson ML, Nelson JC. The test-negative design for estimating influenza vaccine effectiveness. *Vaccine* 2013; **31**(17): 2165-8.

25. Sullivan SG, Tchetgen Tchetgen EJ, Cowling BJ. Theoretical Basis of the Test-Negative Study Design for Assessment of Influenza Vaccine Effectiveness. *Am J Epidemiol* 2016; **184**(5): 345-53.

26. Centers for Disease Control and Prevention. CDC updates travel guidance for fully vaccinated people. <https://www.cdc.gov/media/releases/2021/p0402-travel-guidance-vaccinated-people.html>. Published April 2, 2021. Accessed April 13, 2023.

27. Occupational Safety and Health Administration. Employer Rights and Responsibilities Following a Federal OSHA Inspection. U.S. Department of Labor. <https://www.osha.gov/sites/default/files/publications/OSHA4159.pdf>. Accessed May 10, 2023.

28. Government of Canada. Testing of vaccinated populations. Public Health Agency of Canada. <https://www.canada.ca/en/public-health/services/diseases/coronavirus-disease-covid-19/testing-screening-contact-tracing/testing-vaccinated-populations.html>. Published August 16, 2021. Updated July 22, 2023. Accessed May 10, 2023.
