# Supplementary material for: Correction to: Impact of quitline services on tobacco cessation: an application of modern epidemiologic methods
Source: Am J Epidemiol. 2026 Sep 28;195(11):2986. doi: 10.1093/aje/kwag207 (PMC13733518; doi:10.1093/aje/kwag207)
Supplement: Supplementary file 1 [file web_material_kwag207.docx]

Supplementary Material

**Impact of Quitline Services on Tobacco Cessation: An Application of Modern Epidemiological Methods**

**Authors**: Ami E. Sedani, Summer G. Frank-Pearce, Sixia Chen, Jennifer D. Peck, Janis E. Campbell, Ann F. Chou, and Laura A. Beebe

**Contents**

[Appendix S1: Methods	2](#_Toc167350496)

[Oklahoma Tobacco Helpline	2](#_Toc167350497)

[Study Population	2](#_Toc167350498)

[Covariates	4](#_Toc167350501)

[References	5](#_Toc167350502)

[TABLES & FIGURES	6](#_Toc167350503)

[TABLES	6](#_Toc167350504)

[Table S1. Quitline service intensity levels.	6](#_Toc167350505)

[Table S2. Summary statistics (diagnostics) for the distribution of:	7](#_Toc167350506)

[Table S3. Results of primary analysis and sensitivity analysis methods estimating risk ratios.	8](#_Toc167350507)

[Table S4. Summary statistics (diagnostics) for the distribution of the inverse probability weights.	9](#_Toc167350508)

[Table S5. Comparison of frequency of baseline variables, before and after application of IPCW.	10](#_Toc167350509)

[Table S6. Comparison of frequency of baseline variables of OTH participants who registered for low intensity services and high intensity services, before and after application of IPTW.	12](#_Toc167350510)

[Table S7. Quit rates by participant characteristics before and after weighting.	15](#_Toc167350511)

[FIGURES	17](#_Toc167350512)

[Figure S1. Simplified causal DAG depicting the association between quitline interventions and cessation a) before and b) after IPCW.	17](#_Toc167350513)

[Figure S2. Simplified causal DAG depicting the association between quitline interventions and cessation after IPCTW weighting.	18](#_Toc167350514)

[Figure S3. Quit rates by service intensity and analysis method.	19](#_Toc167350515)

# Appendix S1: Methods

## Oklahoma Tobacco Helpline

The OTH consistently ranks in the top 20% for reach, tobacco control program investment, and quit rates, following best practices in quitline operations. Available evidence-based cessation services include behavioral coaching (e.g., telephone counseling [helpline], web-based assistance, text messages, emails, a Quit Guide, and nicotine replacement therapy (e.g., nicotine patch, gum, lozenge; [NRT]). Coaching calls utilize motivational interviewing and cognitive-behavioral therapy techniques, guided by the Theory of Planned Behavior framework. At the time of this study, the OTH offered varying levels of intervention as determined by program enrollment including single-call, multiple-call, individual services, and WebCoach programs. Eligibility for these programs was determined by an individual’s health insurance status. Individuals with private insurance were only eligible for the single-call telephone intervention with two weeks of NRT. State employees with HealthChoice were eligible for the multiple call program and up to 12 weeks of NRT. Uninsured individuals were eligible for the multiple call program with up to eight weeks of free NRT, and Medicaid beneficiaries received the multiple call program plus a two-week supply of NRT. Additionally, individuals who were pregnant received a 10-call Helpline intervention specifically designed for this population. Tobacco users with behavioral health conditions were offered a specialized protocol that included seven proactive calls with a coach and up to 12 weeks of combination NRT.

## Study Population

Time zero is when each eligible participant registers (i.e., first contact), and follow-up is at 7 months. The 7-month timeframe allows for one month of treatment and a 6-month measurement from the approximated end of treatment. All participants were using one or more tobacco product(s) at registration. To be included in the follow-up sampling frame, participants were required to have completed at least one coaching call or received 2 weeks of NRT.

### Inclusion

- At least 18 years of age.
- Current tobacco user (past 30 days).
- Ready to quit tobacco.
- Able to speak and read English.
- Not currently pregnant.
- Reside in Oklahoma.

### Exclusion

- Qualify for more robust benefits (health insurance or behavioral health qualification; pregnant).
- Not thinking about quitting tobacco at this time.
- Quit more than 6 months ago.

## Covariates

There is no single best indicator of socioeconomic status (SES) for tobacco research thus it is recommend that measurement decisions be guided by their relevance to the study population, questions and outcomes.^1^ We included income as a proxy for SES, and acknowledge that income does not include all assets (e.g., wealth, health insurance coverage, disability benefits, etc.).^1-3^ We incorporated self-designated race and ethnicity which may represent being in a historically marginalized group thus experiencing interpersonal systemic and structural racism.^1, 4-6^ We combined the two concepts of race and ethnicity as recommended due to the high nonresponse rates among Hispanic/Latino individuals when using separate questions.^7^ One well-established place-based characteristic that contributes to tobacco-related health disparities is rural-urban status.^8^ Rural–urban health disparities are multidimensional, likely reflecting many variables (reduced access to health care and public health services, differences in built environment). We categorized geographic location type into three categories based on participant’s county using the rural-urban commuting area (RUCA) codes from the United States Department of Agriculture (USDA), which uses measures of population density, urbanization, and daily commuting patterns.^9, 10^

Time after waking to first tobacco use was used as a surrogate for nicotine dependence. The best-known dependence measures are those developed by Fagerström and colleagues (e.g., the original Fagerström Tolerance Questionnaire, the Fagerström Test for Nicotine Dependence, the Heaviness of Smoking Index).^11, 12^ Findings have shown that time after waking to first tobacco use appears to be the most predictive measure of dependence from the Fagerström Test for Nicotine Dependence.^13-15^ Specifically, those who use tobacco within five minutes of waking may have greater difficulty quitting compared to longer periods after waking.

## References

1.	Weinberger AH, Steinberg ML, Mills SD, et al. Assessing Sex, Gender Identity, Sexual Orientation, Race, Ethnicity, Socioeconomic Status, and Mental Health Concerns in Tobacco Use Disorder Treatment Research: Measurement Challenges and Recommendations From a Society for Research on Nicotine and Tobacco Pre-conference Workshop. *Nicotine and Tobacco Research*. 2022;24(5):643-653.

2.	Shavers VL. Measurement of socioeconomic status in health disparities research. *Journal of the national medical association*. 2007;99(9):1013.

3.	Turrell G. Income non-reporting: implications for health inequalities research. *Journal of epidemiology & community health*. 2000;54(3):207-214.

4.	Bailey ZD, Feldman JM, Bassett MT. How structural racism works—racist policies as a root cause of US racial health inequities. Mass Medical Soc; 2021. p. 768-773.

5.	Bailey ZD, Krieger N, Agénor M, Graves J, Linos N, Bassett MT. Structural racism and health inequities in the USA: evidence and interventions. *The lancet*. 2017;389(10077):1453-1463.

6.	Dean LT, Thorpe Jr RJ. What structural racism is (or is not) and how to measure it: clarity for public health and medical researchers. *American Journal of Epidemiology*. 2022;191(9):1521-1526.

7.	Mathews K, Phelan J, Jones NA, et al. National Content Test: Race and ethnicity analysis report. *US Department of Commerce, Economics and Statistics Administration, US Census Bureau*. 2015;

8.	Doogan N, Roberts M, Wewers M, et al. A growing geographic disparity: rural and urban cigarette smoking trends in the United States. *Preventive medicine*. 2017;104:79-85.

9.	Center WRHR. RUDA Data: Using RUCA Data. Accessed November 19, 2019, 2019. <http://depts.washington.edu/uwruca/ruca-uses.php>

10.	Bureau USC. Metropolitan and Micropolitan. Updated July 5, 2022. 2022. <https://www.census.gov/programs-surveys/metro-micro.html>

11.	Fagerström K-O. Measuring degree of physical dependence to tobacco smoking with reference to individualization of treatment. *Addictive behaviors*. 1978;3(3-4):235-241.

12.	Heatherton TF, Kozlowski LT, Frecker RC, Fagerstrom KO. The Fagerström test for nicotine dependence: a revision of the Fagerstrom Tolerance Questionnaire. *British journal of addiction*. 1991;86(9):1119-1127.

13.	Fagerstrom K. Time to first cigarette; the best single indicator of tobacco dependence? *Monaldi Archives for Chest Disease*. 2003;59(1):91-94.

14.	Baker TB, Piper ME, McCarthy DE, et al. Time to first cigarette in the morning as an index of ability to quit smoking: implications for nicotine dependence. *Nicotine & Tobacco Research*. 2007;9(Suppl_4):S555-S570.

15.	Borland R, Yong H-H, O'connor R, Hyland A, Thompson M. The reliability and predictive validity of the Heaviness of Smoking Index and its two components: findings from the International Tobacco Control Four Country study. *Nicotine & Tobacco Research*. 2010;12(suppl_1):S45-S50.

# TABLES & FIGURES

## TABLES

### Table S1. Quitline service intensity levels.

| **INTERVENTION ARM** | **INTENSITY LEVEL** | **COMPLETED CALLS** | **NRT SUPPLIED (WEEKS)** |
| --- | --- | --- | --- |
| **Active Comparator: Low**  [(n=1,704) 36.29%] | **Level 1** (n=1,704) | 0 | 2 |
|  |  | 1 | 0 |
| **Experimental: High**  [(n=2,991) 63.71%] | **Level 2**  (n=1,160) | 0 | 4-6 |
|  |  | 1 | 2 |
|  |  | 2 | 0 |
|  | **Level 3**  (n=811) | 0 | ≥ 8 |
|  |  | 1 | 4-6 |
|  |  | 2 | 2 |
|  |  | >2 | 0 |
|  | **Level 4**  (n=1,020) | 1 | ≥ 8 |
|  |  | 2 | ≥ 4 |
|  |  | >2 | ≥ 2 |

### Table S2. Summary statistics (diagnostics) for the distribution of:

a. the inverse probability of censoring weights

|  | **Stabilized** | **Truncation** | | | **Raking** |
| --- | --- | --- | --- | --- | --- |
|  |  | **1, 99** | **5, 95** | **10, 90** |  |
| **Mean** | 1.00 | 2.10 | 2.08 | 2.07 | 2.11 |
| **STD** | 0.15 | 0.39 | 0.32 | 0.26 | 0.42 |
| **Minimum** | 0.63 | 1.48 | 1.59 | 1.68 | 1.40 |
| **Maximum** | 1.68 | 3.40 | 2.97 | 2.68 | 4.18 |
| **1 percentile** | 0.72 | 1.52 | 1.61 | 1.69 | 1.48 |
| **Median** | 0.97 | 2.02 | 2.02 | 2.02 | 2.02 |
| **99 percentile** | 1.41 | 3.24 | 2.91 | 2.66 | 3.40 |
| **RR (95% CI)** | 1.14  (1.00, 1.30) | 1.15  (1.02, 1.32) | 1.25 (1.08, 1.45) | 1.27 (1.08, 1.49) | 1.14  (1.00, 1.30) |

b. the inverse probability of censoring x treatment weights

|  | **Stabilized** | **Truncation** | | | **Raking** |
| --- | --- | --- | --- | --- | --- |
|  |  | **1, 99** | **5, 95** | **10, 90** |  |
| **Mean** | 1.00 | 4.18 | 4.06 | 3.93 | 4.23 |
| **STD** | 0.25 | 1.96 | 1.71 | 1.47 | 2.06 |
| **Minimum** | 0.58 | 1.95 | 2.18 | 2.33 | 1.77 |
| **Maximum** | 2.13 | 9.79 | 8.42 | 7.58 | 11.23 |
| **1 percentile** | 0.63 | 2.03 | 2.21 | 2.33 | 1.96 |
| **Median** | 0.95 | 3.43 | 3.43 | 3.43 | 3.43 |
| **99 percentile** | 1.72 | 9.34 | 8.20 | 7.55 | 9.88 |
| **RR (95% CI)** | 1.23  (1.08, 1.41) | 1.21  (1.06, 1.38) | 1.14  (1.00, 1.32) | 1.09  (0.95, 1.27) | 1.23  (1.08, 1.41) |
| **Abbreviations:** Std, Standard Deviation. | | | | | |

### Table S3. Results of primary analysis and sensitivity analysis methods estimating risk ratios.

|  | **Primary Analysis** | | | |  | **Sensitivity Analysis** | | | |
| --- | --- | --- | --- | --- | --- | --- | --- | --- | --- |
|  | **RR** | **SE** | **95% CI** | **p-value** |  | **RR** | **SE** | **95% CI** | **p-value** |
| **IPCW** | 1.1437 | 0.0760 | 1.0041, 1.3027 | 0.0432 |  | 1.1427 | 0.0760 | 1.0030, 1.3019 | 0.0449 |
| **IPTW** | 1.2684 | 0.0879 | 1.1073, 1.4529 | 0.0006 |  | 1.2704 | 0.0883 | 1.1087, 1.4558 | 0.0006 |
| **IPTCW** | 1.2300 | 0.0841 | 1.0758, 1.4064 | 0.0025 |  | 1.2295 | 0.0842 | 1.0750, 1.4062 | 0.0026 |

**Abbreviations:** IPCW, Inverse Probability Censoring Weight; IPTW, Inverse Probability of Treatment Weight; IPTCW, Inverse Probability of Treatment and Censoring Weight; RR, Risk Ratio; CI, Confidence Intervals; SE, Standard Error.

Primary analysis adjusted for:

(IPCW) intervention group, age, race/ethnicity, health insurance plan, and geographic residence

(IPTW) age, health insurance plan, and income.

Sensitivity Analysis adjusted for: Intervention group (IPCW only), age, gender, race/ethnicity, income, health insurance plan, geographic residence, time after waking to first tobacco use.

IPTCW= IPCW*IPTW.

### Table S4. Summary statistics (diagnostics) for the distribution of the inverse probability weights.

|  | **IPCW** | **IPTW** | **IPCTW** |
| --- | --- | --- | --- |
| **Mean** | 2.11 | 1.94 | 4.22 |
| **Standard Deviation** | 0.42 | 0.75 | 2.05 |
| **Minimum** | 1.40 | 1.21 | 1.75 |
| **Maximum** | 4.18 | 5.95 | 11.99 |
| **1 percentile** | 1.48 | 1.25 | 1.96 |
| **Median** | 2.02 | 1.67 | 3.40 |
| **99-percentile** | 3.40 | 4.56 | 9.79 |
| **RR (95% CI)** | 1.14 (1.00, 1.30) | 1.27 (1.07, 1.45) | 1.23 (1.08, 1.41) |

### Table S5. Comparison of frequency of baseline variables, before and after application of IPCW.

| **CHARACTERISTICS** | **Total Analytic Sample (n= 4,695)** | **Complete Case (n= 2,222)** | **IPCW Sample** |
| --- | --- | --- | --- |
| **Mean Age at Registration** | 48.29 ± 14.95 | 50.13 ± 14.74 | 48.33 ± 25.57 |
| **Age Category** |  |  |  |
| 18-24 | 232 (4.94) | 82 (3.69) | 225.54 (4.81) |
| 25-34 | 805 (17.15) | 319 (14.36) | 787.27 (16.78) |
| 35-44 | 954 (20.32) | 430 (19.35) | 978.47 (20.85) |
| 45-54 | 916 (19.51) | 432 (19.44) | 904.41 (19.27) |
| 55-64 | 1,071 (22.81) | 568 (25.56) | 1,097.36 (23.39) |
| 65+ | 717 (15.27) | 391 (17.60) | 699.46 (14.91) |
| **Gender** |  |  |  |
| Female | 2,631 (56.04) | 1,273 (57.29) | 2,678.65 (57.08) |
| Male | 2,064 (43.96) | 949 (42.71) | 2,013.85 (42.92) |
| **Self-Designated Race/Ethnicity** |  |  |  |
| NH White | 3,307 (70.44) | 1,512 (68.05) | 3,305.01 (70.43) |
| NH Black or African American | 385 (8.20) | 222 (9.99) | 383.59 (8.17) |
| NH AI/AN | 446 (9.50) | 216 (9.72) | 440.02 (9.38) |
| NH Other | 354 (7.54) | 178 (8.01) | 359.40 (7.66) |
| Hispanic | 203 (4.32) | 94 (4.23) | 204.48 (4.36) |
| **Annual Income** |  |  |  |
| Less than $10,000 | 1,448 (30.84) | 674 (30.33) | 1443.40 (30.76) |
| $10,000- 14,999 | 787 (16.76) | 360 (16.20) | 741.52 (15.80) |
| $15,000- 19,999 | 530 (11.29) | 257 (11.57) | 553.00 (11.78) |
| $20,000- 24,999 | 450 (9.58) | 222 (9.99) | 449.21 (9.57) |
| $25,000- 34,999 | 509 (10.84) | 236 (10.62) | 531.15 (11.32) |
| $35,000- 49,999 | 468 (9.97) | 227 (10.22) | 473.31 (10.09) |
| $50,000 or more | 503 (10.71) | 246 (11.07) | 500.91 (10.67) |
| **Health Insurance** |  |  |  |
| Private | 1,008 (21.47) | 496 (22.32) | 1,012.74 (21.58) |
| Medicaid | 923 (19.66) | 437 (19.67) | 911.16 (19.42) |
| Medicare | 1,019 (21.70) | 533 (23.99) | 1,017.72 (21.69) |
| Other Public | 227 (4.83) | 123 (5.54) | 228.99 (4.88) |
| Uninsured | 1,518 (32.33) | 633 (28.49) | 1,521.89 (32.43) |

| **CHARACTERISTICS** | **Total Analytic Sample (n= 4,695)** | **Complete Case (n= 2,222)** | **IPCW Sample** |
| --- | --- | --- | --- |
| **Geographic Residence** |  |  |  |
| Rural | 755 (16.08) | 323 (14.54) | 751.05 (16.01) |
| Micropolitan | 1,017 (21.66) | 492 (22.14) | 1,011.67 (21.56) |
| Metropolitan | 2,923 (62.26) | 1,407 (63.32) | 2,929.78 (62.44) |
| **Past 30-Day Tobacco Product** |  |  |  |
| Multiple tobacco products | 516 (10.99) | 221 (9.95) | 504.33 (10.75) |
| Cigarettes | 3,665 (78.06) | 1,742 (78.40) | 3,636.55 (77.5) |
| Smokeless | 353 (7.52) | 175 (7.88) | 372.07 (7.93) |
| Other | 161 (3.43) | 84 (3.78) | 179.55 (3.83) |
| **Time After Waking to First Tobacco Use** |  |  |  |
| Within 5 minutes | 2,199 (46.84) | 981 (44.15) | 2,100.95 (44.77) |
| 6-30 minutes | 1,663 (35.42) | 828 (37.26) | 1,735.54 (36.99) |
| 31-60 minutes | 494 (10.52) | 247 (11.12) | 518.81 (11.06) |
| More than 60 minutes | 339 (7.22) | 166 (7.47) | 337.21 (7.19) |
| **Intervention Intensity** |  |  |  |
| Low | 1,704 (36.29) | 683 (30.74) | 1,701.77 (36.27) |
| High | 2,991 (63.71) | 1,539 (69.26) | 2,990.73 (63.73) |
| **Notes**: Past 30-Day Tobacco Product variable does not include electronic cigarettes (with or without nicotine) and other vaping devices  Inverse Probability Censoring Weight (IPCW) created with adjustment for intervention, age, race/ethnicity, health insurance plan, and geographic residence. | | | |

### Table S6. Comparison of frequency of baseline variables of OTH participants who registered for low intensity services and high intensity services, before and after application of IPTW.

|  | **BEFORE WEIGHTING** | | **AFTER WEIGHTING,**  **IPTW** | |
| --- | --- | --- | --- | --- |
|  |  |  |  |  |
|  | **Low Intensity** | **High Intensity** | **Low Intensity** | **High Intensity** |
| **Age Category** |  |  |  |  |
| 18-24 | 114 (6.69) | 118 (3.95) | 80.87 (4.20) | 84.35 (3.53) |
| 25-34 | 357 (20.95) | 448 (14.98) | 265.21 (13.79) | 362.81 (15.17) |
| 35-44 | 382 (22.42) | 572 (19.12) | 374.96 (19.49) | 466.62 (19.51) |
| 45-54 | 338 (19.84) | 578 (19.32) | 359.80 (18.70) | 465.82 (19.48) |
| 55-64 | 327 (19.19) | 744 (24.87) | 499.98 (25.99) | 597.36 (24.98) |
| 65+ | 186 (10.92) | 531 (17.75) | 342.96 (17.83) | 414.30 (17.33) |
| **Gender** |  |  |  |  |
| Female | 933 (54.75) | 1,698 (56.77) | 1,112.96 (57.85) | 1,112.96 (57.85) |
| Male | 771 (45.25) | 1,293 (43.23) | 810.82 (42.15) | 810.82 (42.15) |
| **Race/Ethnicity** |  |  |  |  |
| NH White | 1,220 (71.60) | 2,087 (69.78) | 1,321.93 (68.72) | 1,615.13 (67.54) |
| NH Black or African American | 118 (6.92) | 267 (8.93) | 192.51 (10.01) | 243.12 (10.17) |
| NH AI/AN | 172 (10.09) | 274 (9.16) | 186.92 (9.72) | 232.88 (9.74) |
| NH Other | 131 (7.69) | 223 (7.46) | 159.59 (8.30) | 184.35 (7.71) |
| Hispanic | 63 (3.70) | 140 (4.68) | 62.83 (3.27) | 115.77 (4.84) |

|  | **BEFORE WEIGHTING** | | **AFTER WEIGHTING^a^** | |
| --- | --- | --- | --- | --- |
|  |  |  |  |  |
|  | **Low Intensity** | **High Intensity** | **Low Intensity** | **High Intensity** |
| **Annual Income** |  |  |  |  |
| Less than $10,000 | 495 (29.05) | 972 (32.50) | 583.48 (30.33) | 583.48 (30.33) |
| $10,000- 14,999 | 255 (14.96) | 529 (17.69) | 281.53 (14.63) | 281.53 (14.63) |
| $15,000- 19,999 | 161 (9.45) | 361 (12.07) | 260.28 (13.53) | 260.28 (13.53) |
| $20,000- 24,999 | 163 (9.57) | 287 (9.60) | 179.35 (9.32) | 179.35 (9.32) |
| $25,000- 34,999 | 212 (12.44) | 293 (9.80) | 227.58 (11.83) | 227.58 (11.83) |
| $35,000- 49,999 | 187 (10.97) | 281 (9.39) | 184.91 (9.61) | 184.91 (9.61) |
| $50,000 or more | 231 (13.56) | 268 (8.96) | 206.64 (10.74) | 206.64 (10.74) |
| **Insurance** |  |  |  |  |
| Private | 443 (26.00) | 565 (18.89) | 446.44 (23.21) | 535.78 (22.41) |
| Medicaid | 300 (17.61) | 623 (20.83) | 355.48 (18.48) | 480.97 (20.11) |
| Medicare | 268 (15.73) | 751 (25.11) | 446.52 (23.21) | 569.33 (23.81) |
| Other Public | 90 (5.28) | 137 (4.58) | 114.52 (5.95) | 128.38 (5.37) |
| Uninsured | 603 (35.39) | 915 (30.59) | 560.82 (29.15) | 676.80 (28.30) |
| **Geographic Residence** |  |  |  |  |
| Rural | 278 (16.31) | 477 (15.95) | 272.86 (14.18) | 344.94 (14.43) |
| Micro | 383 (22.48) | 634 (21.20) | 447.5 (23.26) | 515.55 (21.56) |
| Metro | 1,043 (61.21) | 1,880 (62.86) | 1,203.41 (62.55) | 1,530.77 (64.02) |

|  | **BEFORE WEIGHTING** | | **AFTER WEIGHTING^a^** | |
| --- | --- | --- | --- | --- |
|  |  |  |  |  |
|  | **Low Intensity** | **High Intensity** | **Low Intensity** | **High Intensity** |
| **Time to first use of tobacco** |  |  |  |  |
| Within 5 minutes | 776 (45.54) | 1423 (47.58) | 809.01 (42.05) | 809.01 (42.05) |
| 6-30 minutes | 611 (35.86) | 1052 (35.17) | 761.28 (39.57) | 761.28 (39.57) |
| 31-60 minutes | 198 (11.62) | 296 (9.90) | 220.53 (11.46) | 220.53 (11.46) |
| More than 60 minutes | 119 (6.98) | 220 (7.36) | 132.97 (6.91) | 132.97 (6.91) |
| **Past 30-Day Tobacco Product** |  |  |  |  |
| Multiple tobacco products | 197 (11.56) | 319 (10.67) | 220.04 (11.44) | 220.04 (11.44) |
| Cigarette | 1,296 (76.06) | 2,369 (79.2) | 1484.50 (77.17) | 1,484.50 (77.17) |
| Smokeless | 142 (8.33) | 211 (7.05) | 149.96 (7.80) | 149.96 (7.80) |
| Other | 69 (4.05) | 92 (3.08) | 69.28 (3.60) | 69.28 (3.60) |

^a^ Weighted using IPTW.

Inverse Probability of Treatment Weight (IPTW) created with adjustment for age, health insurance plan, and income.

### Table S7. Quit rates by participant characteristics before and after weighting.

|  | **QUIT RATES** | |
| --- | --- | --- |
|  | **COMPLETE CASE** | **WEIGHTED^a^** |
| **Age Category** |  |  |
| 18-24 | 52.44 (41.63, 63.25) | 51.81 (40.68, 62.95) |
| 25-34 | 39.50 (34.13, 44.86) | 38.51 (32.96, 44.06) |
| 35-44 | 34.42 (29.93, 38.91) | 34.62 (29.84, 39.40) |
| 45-54 | 31.94 (27.55, 36.64) | 30.02 (25.37, 34.68) |
| 55-64 | 30.63 (26.84, 34.42) | 28.89 (24.73, 33.04) |
| 65+ | 37.34 (32.55, 42.13) | 33.24 (27.66, 38.83) |
| **Gender** |  |  |
| Female | 32.29 (29.72, 34.85) | 31.19 (28.37, 34.00) |
| Male | 38.36 (35.26, 41.45) | 37.06 (33.70, 40.42) |
| **Self-Designated Race/Ethnicity** |  |  |
| NH White | 35.71 (33.30, 38.13) | 34.25 (31.62, 36.88) |
| NH Black | 34.23 (27.99, 30.48) | 32.23 (25.49, 38.96) |
| NH AI/AN | 27.78 (21.80, 33.75) | 26.59 (20.23, 32.94) |
| NH Other | 34.83 (27.83, 41.83) | 34.81 (27.20, 42.42) |
| Hispanic | 39.36 (29.49, 49.24) | 40.83 (30.00, 51.65) |
| **Annual Income** |  |  |
| Less Than $10,000 | 34.12 (30.55, 37.70) | 31.49 (27.61, 35.37) |
| $10,000- 14,999 | 31.11 (26.33, 35.89) | 31.41 (25.99, 36.84) |
| $15,000- 19,999 | 28.40 (22.89, 33.92) | 27.77 (21.53, 34.01) |
| $20,000- 24,999 | 36.04 (29.72, 42.35) | 35.00 (28.03, 41.97) |
| $25,000- 34,999 | 34.32 (28.26, 40.38) | 32.75 (26.51, 39.00) |
| $35,000- 49,999 | 39.65 (33.28, 46.01) | 40.77 (33.84, 47.69) |
| $50,000 or More | 44.31 (38.10, 50.52) | 43.44 (36.98, 49.89) |
|  | | |

|  | **QUIT RATES** | |
| --- | --- | --- |
|  | **COMPLETE CASE** | **WEIGHTED^a^** |
| **Health Insurance Status** |  |  |
| Private | 39.31 (35.02, 43.61) | 39.20 (34.73, 43.68) |
| Medicaid | 28.38 (24.15, 32.60) | 27.02 (22.40, 31.65) |
| Medicare | 35.65 (31.58, 39.71) | 32.32 (27.63, 37.01) |
| Other Public | 39.84 (31.19, 48.49) | 39.27 (30.14, 48.40) |
| Uninsured | 34.28 (30.58, 37.98) | 34.02 (30.07, 37.98) |
| **Geographic Residence** |  |  |
| Rural | 36.22 (30.98, 41.46) | 36.09 (30.23, 41.95) |
| Micropolitan | 34.15 (29.96, 38.34) | 32.60 (28.05, 37.15) |
| Metropolitan | 34.83 (32.34, 37.32) | 33.46 (30.77, 36.15) |
| **Time to First Tobacco Use** |  |  |
| Within 5 Minutes | 33.13 (30.18, 36.07) | 31.98 (28.79, 35.18) |
| 6-30 Minutes | 35.14 (31.89, 38.40) | 33.53 (29.99, 37.07) |
| 31-60 Minutes | 40.49 (34.36, 46.61) | 38.83 (32.21, 45.46) |
| More Than 60 Minutes | 35.54 (28.26, 42.82) | 36.97 (28.74, 45.21) |
| **Tobacco Product** |  |  |
| Multiple Tobacco Products | 32.13 (25.97, 38.28) | 32.12 (25.5, 38.73) |
| Cigarettes | 32.32 (30.12, 34.52) | 30.91 (28.52, 33.30) |
| Smokeless | 51.43 (44.02, 58.83) | 49.93 (41.92, 57.95) |
| Other | 60.71 (50.27, 71.16) | 61.99 (50.72, 73.27) |
| **Intervention Intensity** |  |  |
| Low | 31.04 (27.57, 34.51) | 30.21 (26.71, 33.72) |
| High | 36.58 (34.18, 38.99) | 37.16 (34.66, 39.65) |
| **^a^**Weighted using IPTCW.  Inverse Probability of Treatment and Censoring Weight (IPTCW) is IPCW*IPTW, where Inverse Probability Censoring Weight (IPCW) was created with adjustment for intervention, age, race/ethnicity, health insurance plan, and geographic residence, and Inverse Probability of Treatment Weight (IPTW) was created with adjustment for age, health insurance plan, and income. | | |

## FIGURES

### Figure S1. Simplified causal DAG depicting the association between quitline interventions and cessation a) before and b) after IPCW.

A: represents the intervention (quitline services)

Y: represents the outcome (tobacco cessation)

Z: represents the vector of measured baseline covariates

C: represents censoring (responders vs. non-responders)

U: represents a vector of unmeasured common causes of the baseline confounders and the outcome 𝑌

### Figure S2. Simplified causal DAG depicting the association between quitline interventions and cessation after IPCTW weighting.

### Figure S3. Quit rates by service intensity and analysis method.

**Notes**:

χ 2 test was used to calculate the p-value. *Statistically significant at ≤0.05

Selection Bias: Inverse Probability Censoring Weight (IPCW) created with adjustment for intervention, age, race/ethnicity, health insurance status, and geographic residence.

Confounding: Inverse Probability of Treatment Weight (IPTW) created with adjustment for age, health insurance status, and income.

Selection Bias and Confounding: Inverse Probability of Treatment and Censoring Weight (IPTCW) is IPCW*IPTW.
